# Regulation of Adult CNS Axonal Regeneration by the Post-transcriptional Regulator Cpeb1

**DOI:** 10.3389/fnmol.2017.00445

**Published:** 2018-01-12

**Authors:** Wilson Pak-Kin Lou, Alvaro Mateos, Marta Koch, Stefan Klussman, Chao Yang, Na Lu, Sachin Kumar, Stefanie Limpert, Manuel Göpferich, Marlen Zschaetzsch, Christopher Sliwinski, Marc Kenzelmann, Matthias Seedorf, Carlos Maillo, Elena Senis, Dirk Grimm, Radhika Puttagunta, Raul Mendez, Kai Liu, Bassem A. Hassan, Ana Martin-Villalba

**Affiliations:** ^1^Division of Molecular Neurobiology, German Cancer Research Center, Heidelberg, Germany; ^2^Faculty of Biosciences, University of Heidelberg, Heidelberg, Germany; ^3^VIB Center for the Biology of Disease and Center for Human Genetics, VIB and KU Leuven, Leuven, Belgium; ^4^Division of Life Science, State Key Laboratory of Molecular Neuroscience, Hong Kong University of Science and Technology, Hong Kong, Hong Kong; ^5^Department of Neuroregeneration, University Hospital Heidelberg, Heidelberg, Germany; ^6^Division of Molecular Biology of the Cell I, German Cancer Research Center, Heidelberg, Germany; ^7^Zentrum für Molekulare Biologie, University of Heidelberg, Heidelberg, Germany; ^8^Translational Control of Cell Cycle and Differentiation, Institute for Research in Biomedicine, Barcelona, Spain; ^9^Virus Host Interaction, Heidelberg University Hospital, Center for Infectious Diseases/Virology, Cluster of Excellence CellNetworks, BioQuant, Heidelberg, Germany; ^10^Center of Systems Biology and Human Health, School of Science and Institute for Advanced Study, Hong Kong University of Science and Technology, Hong Kong, Hong Kong; ^11^Sorbonne Universités, UPMC Univ Paris 06, Institut National de la Santé et de la Recherche Médicale, Centre National de la Recherche Scientifique, Institut du Cerveau et de la Moelle epiniere - Hôpital Pitié-Salpêtrière, Paris, France

**Keywords:** axon regeneration, translation, polysome profiling, motif analysis, CPEB1, spinal cord injuries

## Abstract

Adult mammalian central nervous system (CNS) neurons are unable to regenerate following axonal injury, leading to permanent functional impairments. Yet, the reasons underlying this regeneration failure are not fully understood. Here, we studied the transcriptome and translatome shortly after spinal cord injury. Profiling of the total and ribosome-bound RNA in injured and naïve spinal cords identified a substantial post-transcriptional regulation of gene expression. In particular, transcripts associated with nervous system development were down-regulated in the total RNA fraction while remaining stably loaded onto ribosomes. Interestingly, motif association analysis of post-transcriptionally regulated transcripts identified the cytoplasmic polyadenylation element (CPE) as enriched in a subset of these transcripts that was more resistant to injury-induced reduction at the transcriptome level. Modulation of these transcripts by overexpression of the CPE binding protein, Cpeb1, in mouse and *Drosophila* CNS neurons promoted axonal regeneration following injury. Our study uncovered a global evolutionarily conserved post-transcriptional mechanism enhancing regeneration of injured CNS axons.

## Introduction

Axons of the adult mammalian CNS have a very limited regenerative capacity following injury. After injury, axons rostral to the lesion form retraction bulbs and die back, retracting from the injury site, and those caudal to the lesion undergo Wallerian degeneration (Bernstein and Stelzner, 1983; Bregman et al., 1989). Whereas severed axons were seldom observed to regrow into the lesion, a level of functionality could potentially be recovered by rewiring the circuit. This could be achieved, for example, by growing into the uninjured ventral column after a dorsal hemisection injury to bypass the lesion (Steward et al., 2008), or by sprouting onto unaffected axons of intermediate or propriospinal neurons (Bareyre et al., 2004).

However, the question remains as to why severed axons are unable to regrow into the lesion site. Extrinsic factors in the extracellular matrix of the lesion site with inhibitory effect on regeneration have received a great deal of attention, and many molecules have been identified (Filbin, 2006). However, the limited success obtained by strategies based on neutralizing inhibitory signals within the immediate environment of an injured axon (Côté et al., 2011; Young, 2014), has recently turned the attention to cell intrinsic factors involved in positive and negative modulation of the regenerative response (Liu et al., 2011; Mar et al., 2014). Indeed, the intrinsic ability of axons to regrow toward the lesion was already described early in 1913 by Santiago Ramón y Cajal. Besides the weak and sterile end of axotomized axons set to degenerate, there are active axonal ends, capable of sprouting, for which he termed “bud” or “club of growth”, due to their analogy to the growth cones of embryonic axons. However, this initial attempt to regrow is largely unsuccessful and eventually stops (Cajal et al., 1991). Interestingly, formation of sprouts at the axonal tip of axotomized dorsal root ganglion (DRG) neurons is accompanied by increased expression of regeneration-associated genes (Ylera et al., 2009). Successful regrowth of central DRG axons as induced by a preconditioning peripheral lesion requires the assembly of these regenerating terminal bulbs that are observed 5–7 h following injury (Ylera et al., 2009). Processes like membrane sealing, regulation of proteolytic processes, RNA stability, and local translation are major determinants of successful assembly of a regenerating axonal terminal. Therefore, injured CNS axons do express a programme to regrow in the early post-injury phases but ultimately fail to do so. Consistent with this, it has been shown that an intrinsic pro-regenerative response has to be stimulated before the onset of overt inflammatory response and scar formation (Davies et al., 1997). As a result, studying the early post-injury phase could reveal key information, from which we could learn how neurons behave in a regeneration-permissive state, and more importantly, whether this state could be extended to promote axonal regeneration.

There exists a number of models to study CNS injury, each with different advantages. A dorsal transection to the mouse spinal cord provides a close clinical relevance to spinal cord injury in humans, while being sufficiently reproducible (Lee and Lee, 2013). The optical nerve crush model provides excellent reproducibility when measuring axon regrowth, as the optic nerve is composed largely of axons from retinal ganglion cells (Xue et al., 2016). *In vitro* primary cultures of embryonic neurons have the advantage of easy genetic manipulation. In addition, despite the considerable phylogenetic distance between *Drosophila* and mouse, many phenomena and mechanisms of CNS development and regeneration are conserved between the two species (Hoopfer et al., 2006; MacDonald et al., 2006; Song et al., 2012), including the transient abortive regeneration after CNS injury (Ayaz et al., 2008). This allows application of results from fly genetics to speed up finding and validation of candidate regulator genes for regeneration in mouse models. We made use of these models in the current study to complement one another and thereby identify overarching principles governing axonal de/regeneration across different models and species.

Post-transcriptional regulation of gene expression is of particular importance in neurons, as it allows enhanced spatial and temporal control in locations distant from the cell soma, such as synapses and axonal growth cones (Jung et al., 2012; Holt and Schuman, 2013). Indeed, axonal regeneration in peripheral sensory neurons involves post-transcriptional regulation, with localized translation of specific transcripts at the tip of the injured axon (Hanz et al., 2003; Perlson et al., 2005; Yudin et al., 2008; Yan et al., 2009; Perry et al., 2012). Interestingly, stability and local translation at the injured axon of some of these regeneration-regulating transcripts is mediated via the 3'UTR of the transcripts. For example, in *C. elegans* adult touch neurons, axotomy induces activation of DLK1 which promotes stabilization and local translation of CEBP-1 by modifying its 3′UTR, and leads to robust regeneration (Yan et al., 2009). 3′ UTR-localized elements are known to nucleate the formation of ribonucleoparticle complexes by binding miRNAs and/or RNA-binding proteins, which in turn, through transport/compartmentalization and regulation of mRNA poly(A) tail length, controls translation, and RNA stability (Weill et al., 2012). Some of these motifs include cytoplasmic polyadenylation element (CPE), Pumilio binding element (PBE), Musashi binding element (MBE), and AU-rich elements (AREs) (von Roretz et al., 2011; Charlesworth et al., 2013). CPE is best known for its role in cytoplasmic polyadenylation, where it regulates the length of the poly(A) tail of the transcripts that contain it and thus its translation (Weill et al., 2012; Ivshina et al., 2014). CPE-mediated regulation can be complex, as it could act in conjunction with other cis elements such as PBE, MBE, AREs, or miRNA binding elements whereby the number, arrangement and distance between motifs and availability of their regulators such as kinases or RBPs have varying effects on transport, stability, or translation of the transcript (Piqué et al., 2008; Weill et al., 2012). In the nervous system, Cpeb1 is known to transport transcripts to postsynaptic densities in dendrites, where it promotes their translation upon synaptic activity (Wu et al., 1998; Huang et al., 2003; Udagawa et al., 2012).

We reasoned that the early transient regenerative response might offer key insights into the intrinsic molecular mechanisms that need to be activated for successful regeneration. To this end, we profiled total and polysome-bound RNAs of sham operated (naïve) and acutely-injured spinal cords 9 h following injury—a time when some axons still show regenerative-growth cones and infiltration of the spinal cord by inflammatory cells is barely detectable (Letellier et al., 2010). We find that changes in the two RNA fractions are highly uncoupled, and that uncoupled genes could successfully predict axonal growth regulators in a *Drosophila* model. Presence of the CPE motif in some of these transcripts correlated with resistance to injury-induced decrease of transcript availability. Finally, we show that in a *Drosophila* axonal-injury model and a mouse model of optic nerve crush-injury, axonal regeneration is enhanced upon Cpeb1 overexpression. This study uncovers a highly conserved global switch in neurons that increases availability of regeneration-associated transcripts and thereby enables axonal regeneration following axotomy.

## Material and methods

### Mouse experiments

#### Spinal cord injury

Female C57BL/N mice of 10 weeks of age were used for spinal cord injury. Animals were bred in house at the DKFZ Center for Preclinical Research Facility and housed under standard conditions. Animals were subjected to an established model for partial transection of the spinal cord (Demjen et al., 2004; Stieltjes et al., 2006; Letellier et al., 2010). Briefly, laminectomy was performed at thoracic level T7/8, followed by a 80% dorsal transection of the spinal cord by cutting the spinal cord with iridectomy scissors. Naïve mice were subjected only to laminectomy. Nine hours after the injury, 2.5 cm of spinal cord tissue centering on the lesion site was extracted. Every procedure was conducted in accordance with DKFZ guidelines and approved by the Regierungpräsidium Karlsruhe.

### Translation state array analysis (TSAA)

RNA was isolated from the chosen tissue and segregated into a total RNA fraction and a polysome-bound RNA fraction. Polysome-bound RNAs was isolated via fractionation in sucrose gradient as previously described (Lou et al., 2014), with fractions containing RNA bound to two or more ribosomes collected.

The samples were profiled with Affymetrix arrays (model Mouse430_a2) with RNA input amounts of 5 and 3 μg for total and polysome-bound RNA fractions respectively. Array data is accessible from GEO (GSE92657). Array data corresponding to each fraction were normalized separately, as polysome-bound RNA is a subset of total RNA and standard microarray normalization methods that assume equality of distributions of total intensity between arrays could not be used.

Normalization was performed using the vsn method as implemented in the vsnrma function from the R/bioconductor package vsn (Huber et al., 2002). lts.quantile = 0.5 was used to allow for robust normalization when many genes are differentially expressed. Differential expression was calculated using limma (Smyth, 2004) from R/bioconductor at the level of probesets, the parameter “trend” was set to TRUE for the empirical moderation of standard errors. Probesets with Benjamini-Hochberg false-discovery rates (FDR) <0.05 were considered as differentially expressed. Probesets were then translated to Ensembl gene IDs (Ensembl v72; www.ensembl.org), and those mapping to multiple IDs were excluded from subsequent analysis. For cell marker analysis normalized microarray intensities were averaged over replicates. The expression values for 103 measured marker genes were used to show the composition of samples.

### Gene ontology enrichment

Enrichment analysis of GO biological process categories between up- and down-regulated genes were performed separately for each of the RNA fractions. To this end, up-regulated genes were compared against a background of all differentially expressed genes with the hypergeometric distribution, using GOstats (Falcon and Gentleman, 2007) and annotation from the org.Mm.eg.db v2.14.0 package, both from R/bioconductor. Under-representation of up-regulated genes in a category is equivalent to having an enrichment of down-regulated genes, and is displayed as such.

For the enrichment study of CPE in the mouse and fly genomes, GO annotations from annotation packages org.Mm.eg.db v2.14.0 and org.Dm.eg.db v2.14.0 with experimental evidence code (i.e., EXP, IDA, IPI, IMP, IGI, IEP) were used. The selectiveness is because GO annotations are based on different kinds of evidence including homology, and circularity would occur when comparing results from two different genomes with homology included. To prevent artificially inflating the number of motifs when genes have more than one transcript with common 3′UTR, we performed the enrichment analysis at the level of genes. Transcripts with annotation of transcript biotype as protein coding were translated to Ensembl gene ID and then to Entrez. Genes with only CPE-containing transcripts were compared against all genes (excluding those with both CPE-containing and CPE-free transcripts) with the hypergeometric distribution using GOstats.

Results from the enrichment analyses were visualized using Cytoscape v3.0.2 (Shannon et al., 2003). Results were represented on the GO subnetworks comprising of categories significant in at least one of the comparisons (Benjamini-Hochberg FDR <1e-4 for **Figure 2** and <1e-5 for **Figure 4C**), with each node referring to one GO category. Color represents the direction of enrichment, and the intensity of color and size of the node represent significance (Benjamini-Hochberg BH FDR >0.05 is depicted as white).

### UTR motif analysis

The UTR sequences from transcripts belonging to genes with Ensembl annotation as known and protein coding were obtained from Ensembl v72 and searched for regular expressions representing motifs. Sequences used for each motif (Tian et al., 2005; Piqué et al., 2008; Spasic et al., 2012) are listed in Table S4.

Probesets were translated to Ensembl transcript ID (Ensembl v72), and only probesets mapping a unique transcript were used. Distributions of log2 (fold change) were compared using the Kolmogorov-Smirnov test in R (http://cran.rproject.org/). To represent the expression change profiles for transcripts from specific GO categories, we used GO annotation at the level of transcript downloaded from Ensembl v72 with the R/Bioconductor package biomaRt.

### Western blotting

Western blotting against Cpeb1 protein in spinal cord tissues was performed with standard procedures using an antibody raised in-house by the group of H. Zentgraf. Cell lysates of murine hippocampal HT22 cells transiently over-expressing Cpeb1 were used as positive control.

### Dual fluorescence *in Situ* hybridization (FISH) and immunohistochemical staining

FISH was performed using RNAscope Fluorescent Multiplex Kit (Advanced Cell Diagnostics) as per the manufacturer's protocol. Briefly, spinal cords were cryosectioned into 10 μm thick sections. Antigen retrieval for FISH was performed by immersing sections in boiling Target Retrieval solution for 5 min, thereafter washing in 100% ethanol. Protease 3 was added to the sections and incubated at 40°C for 30 min. FISH probes for Cpeb1 (channel C1) and dapB (bacterial gene as negative control, channel C3) were added and incubated at 40°C for 2 h. Sections were then incubated with various amplification solutions according to the standard manufacturer's protocol.

After FISH was completed, sections were blocked with 3% goat serum and 0.3% Triton X in PBS for 1 h, then incubated with B3-tubulin antibody (Abcam 21057, 1:200) at 4°C overnight, followered by donkey anti-goat Alexa555 antibody (1:500) and DAPI at room temperature for 2 h.

### Optic nerve crush injury

Experimental procedures were performed in compliance with animal protocols approved by the Animal and Plant Care Facility at the Hong Kong University of Science and Technology. C57BL/6 mice of 5–6 weeks of age were anesthetized with ketamine (80 mg/kg) and xylazine (10 mg/kg) and received Meloxicam (1 mg/kg) as analgesia after the surgery. AAV-vectors (serotype 2/2, titer 1 × 10^12^ vg/ml, 2 μl injection volume) expressing either Cpeb1-HA or Gfp under the neuron-specific human synapsin promoter (hSyn) were injected into the left vitreous bodies of 12 mice with a Hamilton microsyringe. Five weeks after vector injection, the optic nerve was gently exposed intraorbitally and crushed with jeweler's forceps (Dumont #5; Fine Science Tools) around 1 mm behind the optic disk. Mice were kept for 2 weeks after injury before tracing. To visualize RGC axons in the optic nerve, 1.5 μl cholera toxin β subunit conjugated with Alexa555 (CTB555, 2 μg/μl, Invitrogen) were injected into the vitreous bodies. Two days after the CTB injection, animals were sacrificed by transcardial perfusion for histology examination. In each mouse, the completeness of optic nerve crush was verified by showing that anterograde tracing did not reach the superior colliculi.

### Immunofluorescence staining of retina and optic nerve and quantifications

Eyes and optic nerves were cryosectioned and examined under an epifluorescence (Nikon, TE2000) or confocal microscope (Zeiss, LSM Meta710). For determination of RGC infection efficiency and expression levels of Cpeb1, retinal sections were stained with anti-HA (Cell Signaling 2367), and anti-Cpeb1 (Proteintech 13274-1-AP) antibodies. Total numbers of RGCs were determined by whole-mount retina staining with mouse anti-Tuj1 antibody (Covance). Twelve images (3 for each quarter, covering peripheral to central regions of the retina) from each retina were captured under a confocal microscope (400X) and Tuj1-positive cells were counted in a blind fashion. To detect traced axons after optic nerve crush, longitudinal sections of optic nerves were serially collected. To analyze the extent of axonal regeneration in the optic nerve, the number of axons that passed through distance d from the lesion site was estimated using the following formula: $\sum {\text{a}}_{\text{d}}=\pi {\text{r}}^{2}\times \left[\text{average axon numbers per mm}/\text{t}\right]$, where r is equal to half the width of the nerve at the counting site, the average number of axons per mm is equal to the average of (axon number)/(nerve width) in 4 sections per animal, and t is equal to the section thickness (8 μm). Axons were manually counted in a blind fashion. An exponential decrease model was fitted to the data and used to compare the starting numbers of axons and the rates of decrease as one moves away from the lesion point.

### Generation and primary culture of Cpeb1 knockout neurons

Primary cultures of cortical neurons were prepared from embryos of Cpeb1^flox/flox^ mice. Cre-mediated excision will remove exon 4 of Cpeb1 and cause a frameshift that affects the phosphorylation site for activation of Cpeb1 as well as its RNA recognition motifs. Cultures were prepared from cortices of E16.5 embryos. Briefly, dissected cortices were digested with 0.05% trypsin for 15 min, triturated with a fire-polished glass pipette and plated on poly-L-lysine coated surfaces. Neurons were cultured in HS-MEM [1x MEM (Thermo Fisher), 10% horse serum, 1.2% glucose, 4 mM L-glutamine, 1 mM sodium pyruvate, 0.22% NaHCO_3_, 100 U/ml penicillin-streptomycin] and infected with serotype 2 AAV encoding CAG-Cre at an MOI of 1 × 10^5^. Twenty-four hours later medium was replaced with N2B27-MEM [1x MEM (Thermo Fisher), 1x N2 supplement, 1x B27 supplement, 0.1% ovalbumin, 0.6% glucose, 2 mM L-glutamine, 1 mM sodium pyruvate, 0.22% NaHCO_3_, 100 U/ml penicillin-streptomycin].

### *In Vitro* regrowth assay

Neurons were cultured on Fluoroblok transwell chambers with PET membranes with 3 μm pores (Millipore) that allow extension of neuronal processes to the underside of the membrane while keeping cell somas on the top. After 7 days in culture, the underside was scraped with sterile cotton swabs to cut the processes. Twenty-four hours after injury, processes were labeled with addition of 1 μM of Calcein AM (BD Biosciences) to the culture medium and imaged with a Zeiss Cell Observer epifluorescence microscope. Images were analyzed and traced with a custom wrote macro in ImageJ in a blind manner. A mixed effects model was used for statistical comparison to account for variability in various levels of the experimental setup.

### *Drosophila* experiments

#### Stocks and genetics

*Drosophila* melanogaster stocks were kept on standard cornmeal media. For tissue specific overexpression of the transgenes, we used the GAL4/UAS system (Brand and Perrimon, 1993). For the genetic screen in development and after injury, pdf-gal4, uasgfp; pdf-gal4, uas2x egfp/cyo flies were kept as a stock and used to drive expression of the various candidate genes, or crossed to wild-type Canton S (CS), in the case of the outgrowth experiments, or to UAS-lacZ, in the case of the injury experiments. Overexpression stocks were obtained from the Bloomington Stock Centre and the PDF-Gal4 line was obtained from P. Taghert. The list of fly lines used can be found in Table S4. All flies were dissected 2–10 days after eclosion.

### *Drosophila* outgrowth and injury assays

To measure axonal outgrowth during development, flies were reared at 25°C and were dissected in fresh PBS. A minimum of 5 fly brains (10 sLNv projections) per genotype were fixed in 4% formaldehyde and stained with an anti-GFP antibody (Molecular Probes; 1:500), according to standard methods (Ayaz et al., 2008). For the axonal regrowth analysis after injury, flies were reared at 18°C (to minimize developmental effects), and shifted to 25°C 1 day prior to injury. Whole brain explants on culture plate inserts were prepared as described (Ayaz et al., 2008; Koch and Hassan, 2012). In brief, culture plate inserts (Milipore) were coated with laminin and polylysine (BD Biosciences). Fly brains were carefully dissected out in a sterile Petri dish containing ice cold Schneider's *Drosophila* Medium (GIBCO), and transferred to one culture plate insert containing culture medium (10 000 U/ml penicillin, 10 mg/ml streptomycin, 10% Fetal Bovine Serum, and 10 μg/ml insulin in Schneider's *Drosophila* Medium (GIBCO). sLNv axonal injury was performed using an ultrasonic microchisel controlled by a powered device (Eppendorf) as described (Ayaz et al., 2008; Koch and Hassan, 2012) and dishes were kept in a humidified incubator at 25°C. Four days later, cultured brains were fixed and immunohistochemical staining was performed as for freshly dissected samples.

### Imaging, morphometric measurements, and statistics

For the outgrowth experiments, brains were visualized under a fluorescent microscope equipped with a GFP filter and classified as having “increased outgrowth,” “reduced outgrowth,” or “no observable effect” according to comparison of sLNv length with that of controls.

For the injury experiments, de novo growth was assessed 4 days after injury by measuring injured sLNv projection that has formed at least one new axonal sprout of a minimum length of 12 μm. The exact injury location was accessed by comparison with axonal projection length at 5 h (where no de novo growth has occurred). Imaging was performed on a Zeiss 500 or 700 confocal microscope and analyzed with Image J. Regenerated length was defined as the de novo axon lengths using the manual tracing tool. Projected distance was defined as the displacement of the axon sprouts from the lesion site, measured in a straight line. All images were analyzed in a blind manner. Statistical comparisons were performed with two-tailed student's *t*-test.

## Results

### Transcriptome and translatome responses to spinal cord injury are extensively uncoupled

**Figure 1:**
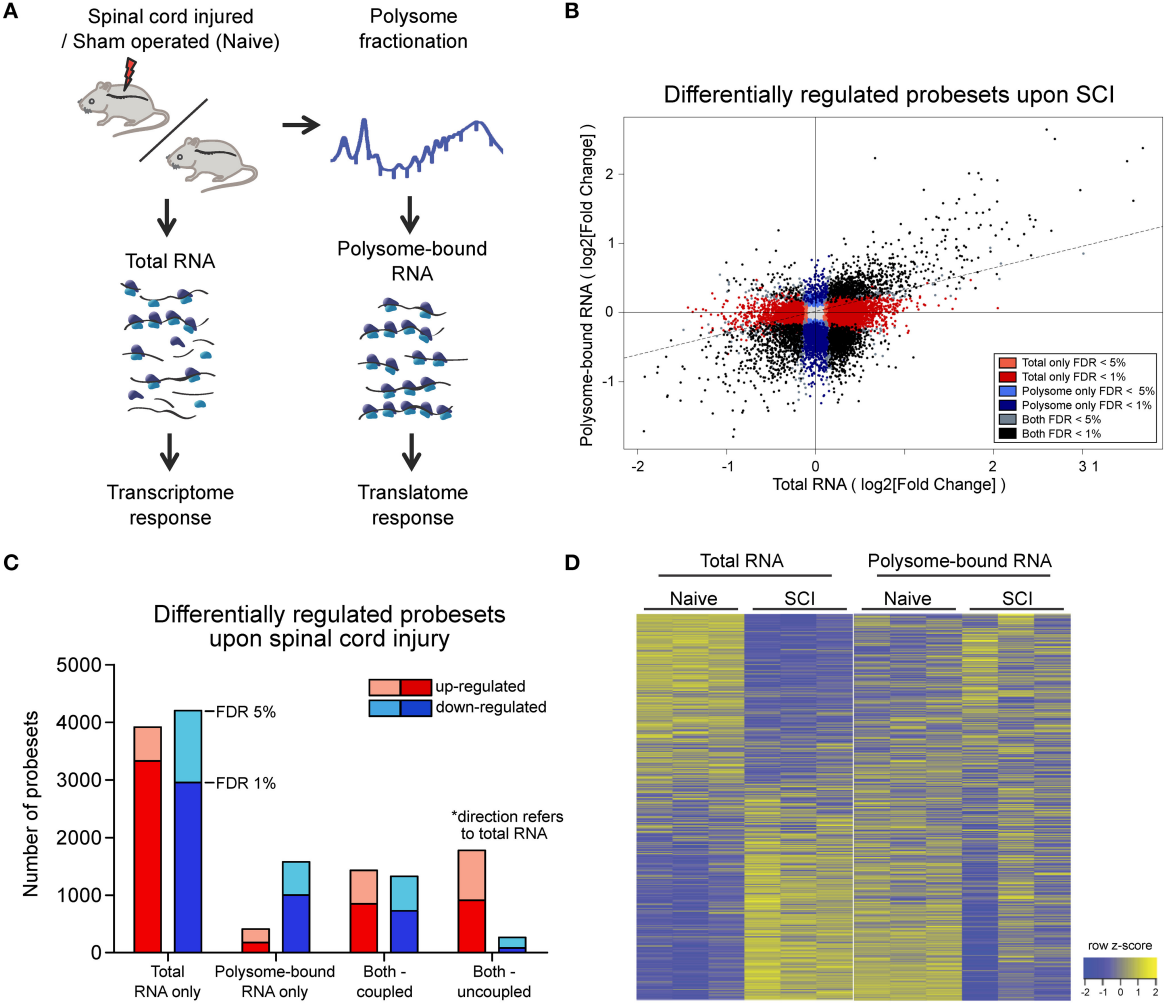
Wide-spread uncoupling of transcriptional and translational responses following spinal cord injury.**(A)** Experimental scheme of simultaneous profile of the transcriptome and translatome. Total or polysome-bound RNA fractions were extracted from naive or injured spinal cords and analyzed by RNA microarray. Three mice were used as biological replicate within each experimental group. **(B)** Scatter plot representation of fold changes of all probesets in total and polysome-bound RNA upon spinal cord injury. **(C)** Number of differentially expressed probesets upon spinal cord injury, grouped according to in which fraction the change occurs and the direction of change. **(D)** Heat map representation of probeset expression as z-score in each sample. Each column represents one biological replicate and each row represents expression of one gene across columns. FDR: Benjamini-Hochberg false discover rate.

To study post-transcriptional regulation in the spinal cord during the abortive regeneration phase following injury, we performed simultaneous profiling of the transcriptome and translatome from injured and naïve mice via translation state array analysis (TSAA). Total and polysome-bound RNAs were extracted from spinal cord tissues of injured and naïve mice 9 h after injury (Figure 1A). This time point was chosen to be within the transient regenerative phase after spinal cord injury, thereby allowing sufficient time for transcriptional and translation changes to take place, under limited immune response conditions (Hausmann, 2003; Trivedi et al., 2006; Schwanhäusser et al., 2011; Gadani et al., 2015). Spinal cord tissue surrounding the injury site was used for RNA profiling, to gain insights into the processes taking place in the axonal ends affected by the injury. In order to obtain sufficient input materials, 2.5 cm of spinal cord tissues were taken. Polysome-bound RNAs were isolated via sucrose gradient fractionation, and fractions containing RNA bound to two or more ribosomes were collected. Samples were subsequently hybridized onto Affymetrix microarrays. Expression changes are listed in Table S1. Notably, correlation plots between arrays shows low variability between replicates, and assessment of expression changes by quantitative real-time PCR (qPCR) largely validated the expression changes obtained from microarray analysis (Figures S1A,B).

Since the chosen tissue not only contains the injured neuronal processes, but also other cellular subtypes, we first analyzed whether the injury would have a major impact on cellular tissue composition at this early time point. To this end we compared the intensities of probesets mapped to marker genes for motor neurons and other local neurons, oligodendrocytes, microglia, precursor cells present in the central canal, and blood-borne cells (Figure S1C, Table S2). The analysis revealed a high correlation between expression patterns of naïve and injured spinal cord (0.97 and 0.99 Pearson correlation for total and polysome-bound RNA fraction, respectively), indicating the absence of major changes in tissue composition upon injury. By contrast, a high proportion of probesets in both total and polysome-bound RNA fractions exhibited significant changes upon injury (Figure 1B). Importantly, for many differentially expressed probesets, the changes in total and polysome-bound RNA fractions do not correlate (Figures 1B–D). A large proportion of changes occur only in the total RNA fraction with no corresponding changes in the polysome-bound RNA fraction. This agrees with previous observations in which stress conditions trigger a general shut down of translation to maximize cell survival (Park et al., 2008; Yamasaki and Anderson, 2008). Differentially regulated genes in the total RNA fraction are similarly distributed between up- and down-regulation. On the other hand, the polysome-bound RNA fraction showed fewer differentially regulated genes than the total RNA fraction, with most of those being down-regulated (Figures 1B,C). The difference in numbers of differentially regulated genes between the total and polysome-bound fractions indicates that the translational response to injury is highly uncoupled from RNA availability. In addition, many genes displayed opposing directions of regulation, suggesting considerable influence of post-transcriptional regulation (Figure 1D).

### Uncoupled genes are functionally clustered and regulate neuronal regeneration

**Figure 2:**
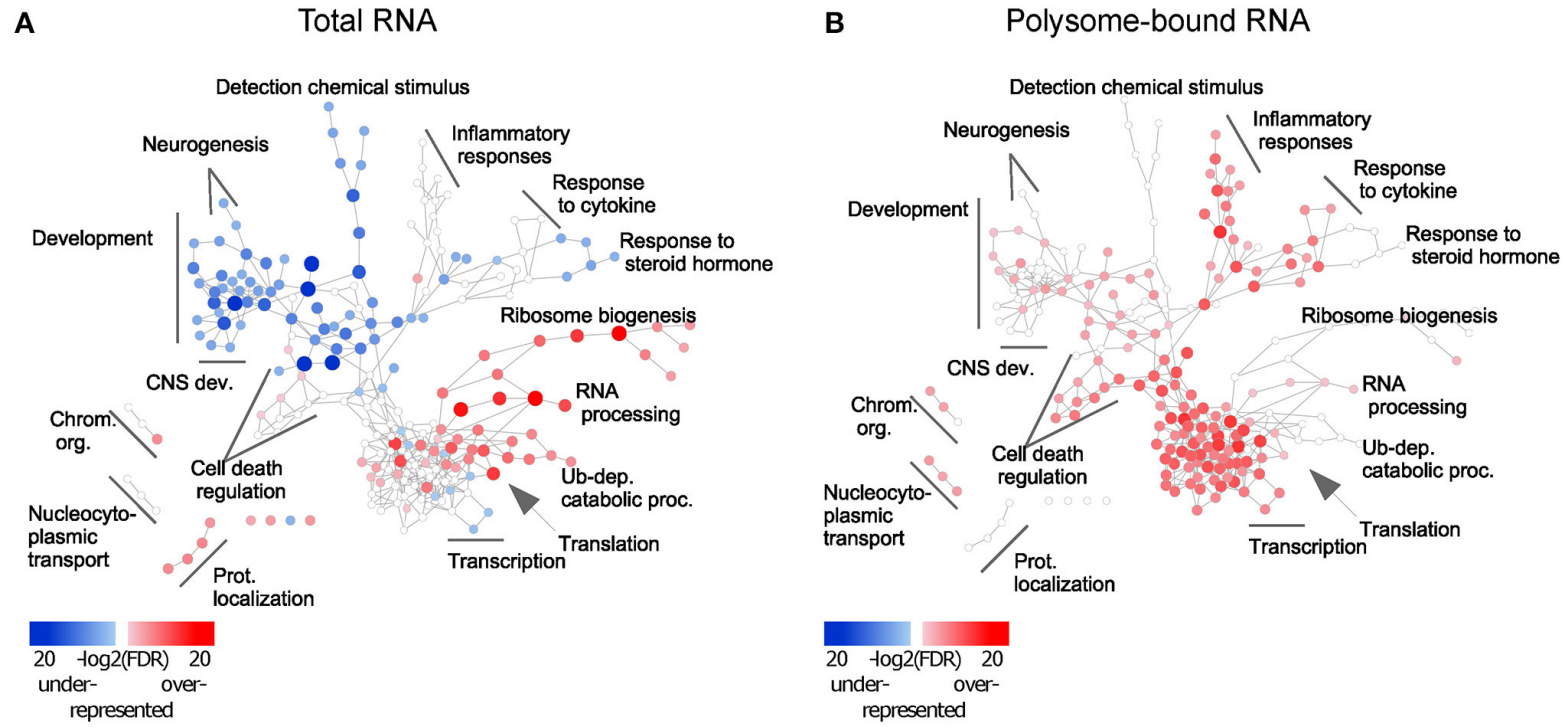
Injury response from total and polysome-bound RNAs is functionally clustered. Gene Ontology enrichment of differentially regulated genes in **(A)** total and **(B)** polysome-bound RNA fractions, represented as a network of GO categories. Enrichment analysis performed as up-regulated genes against all differentially regulated genes. Under-representation is equivalent to an enrichment of down-regulated genes. Color intensity and size of the node represent significance by FDR. Only significantly enriched GO categories (FDR < 1e-4) are shown.

To assess the functional role of the observed uncoupling effect, Gene Ontology (GO) (Ashburner et al., 2011) enrichment analysis was performed and visualized using Cytoscape (Shannon et al., 2003). Enrichment of up- and down-regulated genes was represented as red and blue nodes respectively. In the total-RNA fractions, transcript availability of genes related to translation, RNA processing, protein catabolic processes and protein transport was increased upon injury, but decreased for genes related to CNS development (Figure 2A and Table S3). Excitingly, in the polysome-bound RNA fraction, injury increased ribosome-loading of genes related to regulation of CNS development, as well as cell death, transcription, RNA processing, and immune response (Figure 2B and Table S3). Notably, there were no significantly under-represented categories in the polysome-bound RNA fraction, indicating that the decrease in translation after injury is a general effect and neither directed nor functionally clustered.

Different trends of enrichment were observed for many GO categories between the total and polysome-bound RNA fractions, suggesting that uncoupling serves specific functional purposes. Of particular note, categories related to CNS development, which are decreased in the total RNA fraction, remain stable or enriched in ribosomal-loaded transcripts. This might explain the temporary regeneration observed following injury, which is absent at later stages of the injury response, as existing transcripts from regenerative genes continue to be translated at first, but are not replaced upon eventual degradation.

**Figure 3:**
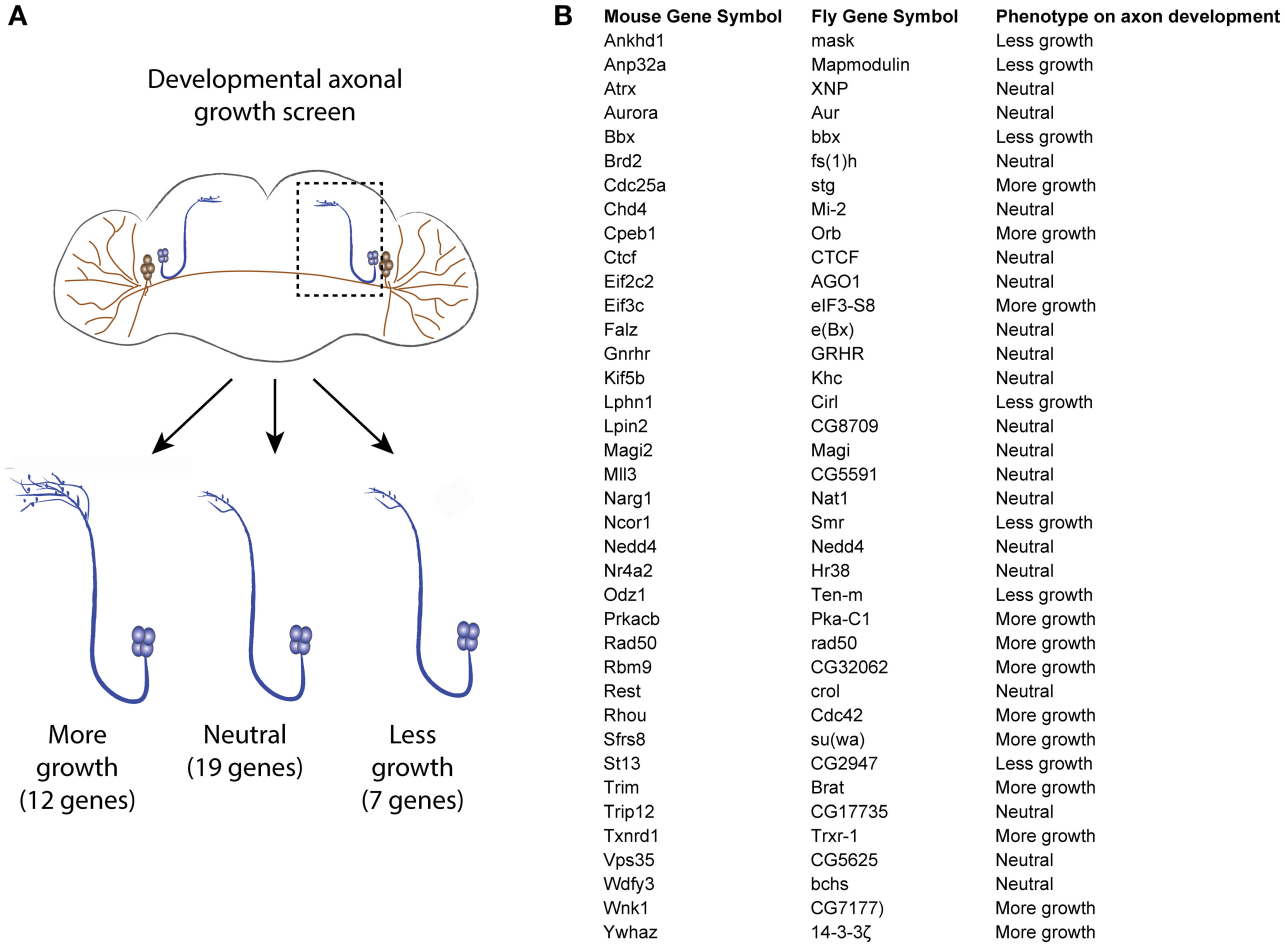
Developmental axonal growth screen in *Drosophila*. **(A)** Experimental scheme and results of the growth screening. Thirty-eight uncoupled genes from the microarray experiment were overexpressed in different fly lines and the effect on sLNv axonal outgrowth was measured. A minimum of five brains were quantified per genotype. **(B)** List of genes tested and the observed effect on axon development.

As the RNA profiling was performed using spinal cord tissues which contain various cell types, it is necessary to investigate if the uncoupled transcripts are of neuronal origin, and thereby affect axonal growth. For this purpose, we turned to *Drosophila*, which allows generation of a large number of neuron-specific transgenic animals in a fast and robust way. Using the UAS-Gal 4 (Brand and Perrimon, 1993), we expressed each of a total of 38 candidate uncoupled genes—for which a fly homolog exists and a UAS line was available—in a fly CNS neuron population called the small ventral lateral neurons (sLNvs) (Figure 3A). Most of the genes tested have reduced transcript availability but stable ribosomal loading in both naïve and injured spinal cord (Table S1). A minimum of 5 brains per genotype was analyzed. Indeed, we found that 19 (50%) of the tested candidates influenced the developmental growth of the sLNv axonal projection, indicating that they have a function in neurons and a role in axonal growth. Amongst them, 12 increased growth, while seven resulted in shorter sLNv projections (Figure 3B and Figure S2).

### Association of 3′UTR motifs with attenuated decrease in transcriptome following spinal cord injury

**Figure 4:**
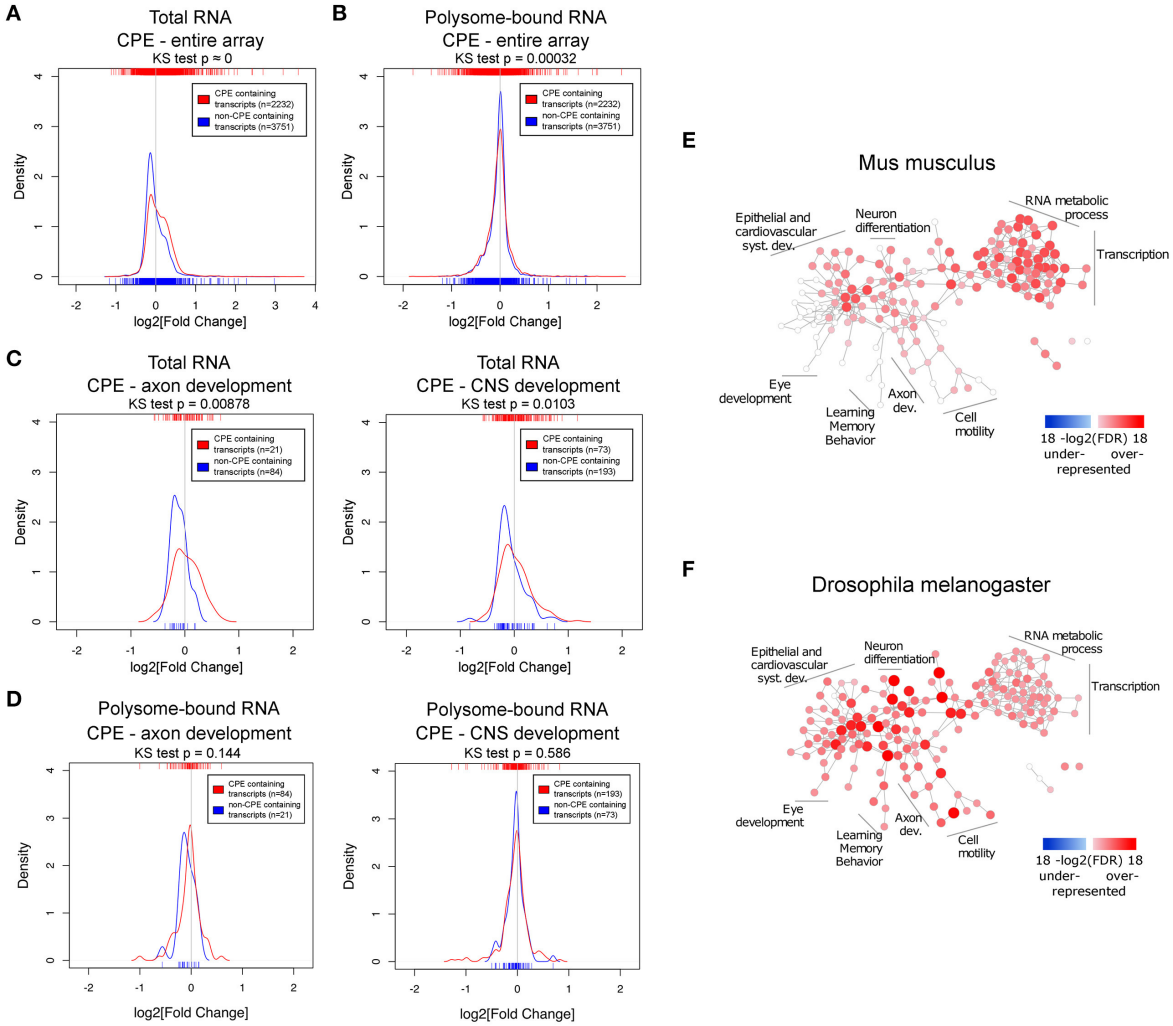
The CPE-motif is enriched in transcripts showing higher positive expression changes following spinal cord injury in the total RNA fraction and in transcripts related to developmental processes. **(A,B)** Density curves showing the distribution of fold changes in expression upon injury of CPE-containing and CPE-free transcripts in **(A)** total and **(B)** polysome-bound RNA from the entire microarray. **(C,D)** Density curves of fold changes in total **(C)** and polysome-bound **(D)** RNA fractions of CPE-containing and CPE-free transcripts of genes associated with GO categories of axon and CNS development. Ticks on top and below the plots represent values of log2 (fold change) of individual transcripts. Distributions were compared with Kolmogorov-Smirnov test. **(E,F)** Enrichment of CPE-containing genes in mouse and *Drosophila* genomes represented as network of GO categories. Intensity of color and size of node represent level of significance. Only GO categories significant in any of the genomes (FDR < 1e-5) are shown.

Next, we asked whether there are common features shared among the uncoupled transcripts, especially since the *Drosophila* experiments suggest that such transcripts are enriched for genes modulating axonal growth. To address this question, we examined the presence of common RNA features. It has been shown that the 3′UTR harbors a myriad of regulatory motifs that regulates stability, intracellular localization and translation of its RNA host (Moore, 2005; Szostak and Gebauer, 2013). Many important neuronal mRNAs such as Map2, Bdnf, β-actin, and Gap43 are regulated via their 3′UTRs (Blichenberg et al., 1999; Lau et al., 2010; Donnelly et al., 2011). This prompted us to investigate the association of 3′UTR motifs in a given transcript with its expression upon injury. Since this has to be performed on the transcript level, only probe sets mapping to a unique transcript were used. We have chosen several 3′UTR motifs to be analyzed based on their published relevance to neuronal functions: cytoplasmic polyadenylation element (CPE) (Si et al., 2010; Darnell and Richter, 2012), Pumilio binding element (PBE) (Vessey et al., 2006; Kaye et al., 2009), and Musashi binding element (MBE) (Nakamura et al., 1994; Hadziselimovic et al., 2014). Hex (hexanucleotide involved in polyadenylation) and AU-rich (AUR) elements (AREs) were also analyzed for their well reported functions in regulating RNA polyadenylation and stability respectively (Colgan and Manley, 1997; Gingerich et al., 2004). The sequences of the motifs used in the analysis are listed in Table S5. To investigate differences in expression changes upon injury relative to motif-free transcripts, we plotted the density curves showing the probability of a data point to have a given log2-fold change, thus reflecting the pattern of distribution of expression of the set of transcripts of interest (Figure 4 and Figures S3, S5).

This analysis revealed that there is a general decrease in levels of transcripts in the total RNA fraction, as indicated by the peaks of the density curves being below zero log2 fold change. However, the density curve of CPE-containing RNAs is notably shifted toward the right side when compared with non-CPE-containing RNAs, and the two curves are significantly different from each other as assessed by Kolmogorov-Smirnov test. (Figure 4A) This shows that CPE-containing transcripts are associated with resistance to injury-induced down-regulation as compared to transcripts devoid of CPE. Notably, this association is also seen when looking specifically at subsets of genes in axon and CNS development GO categories (Figure 4C). In contrast, presence or absence of CPE did not influence injury-induced changes on the level of ribosomal-loading (Figure 4B,D). Likewise, RNA transcripts possessing PBE, MBE, Hex, and AREs were also associated with resistance to injury-induced down-regulation in the total but not in the polysome-bound RNA fractions (Figures S3, S5). However, in contrast to CPE, presence of PBE, MBE and Hex do not affect the levels of RNA in the total fraction of transcripts in axon and CNS development GO categories (Figure S4). We next investigated whether there is a link between AREs and CPE, PBE, MBE, and Hex, and found that they tend to co-occur in the mouse transcriptome. In fact, transcripts containing CPE, PBE, MBE, or Hex tend to also include AREs sequences. In addition, having AREs and one of these motifs is associated with resistance to injury-induced down-regulation in total RNA fraction, suggesting that the AREs motifs might function with the other motifs in a synergistic manner (Figures S5C,D, S6). To ensure that the observed associations between the motifs and fold changes are genuine, the same analysis was performed with the motifs on the 5′UTR or with random motifs. As expected, this control experiment does not show any significant association (Figure S7).

Taken together, the data suggest that CPE, PBE, MBE and Hex confer transcript stability against the global decrease induced by spinal cord injury by increasing RNA stability in conjunction with ARE motifs. Although all the tested 3′UTR motifs are associated with higher resistance to injury-induced down-regulation, only CPE maintained this association in transcripts included in CNS development and axon development GO categories. In addition, CPE-containing genes include validated positive regulators of axonal regeneration such as Cebpβ and c-Jun (Yan et al., 2009; Fontana et al., 2012), which are among the CPE-containing transcripts with the highest up-regulation upon injury in the total RNA fraction (Table S1). Together with the fact that Cpeb1 overexpression in *Drosophila* promoted robust axonal growth of developing sLNvs (Figure 3 and Figure S2B), we chose Cpeb1 for further detailed investigation.

To elucidate whether CPE has a general functional role, GO enrichment analysis was performed on the prevalence of CPE among all protein coding genes of the mouse and fly genomes. Many nervous system development categories were enriched among CPE containing genes in the mouse genome, including neuron projection morphogenesis, axonogenesis and axon guidance (Figure 4E and Table S6). There were no categories found with an under-representation of CPE. Interestingly, almost all categories in the mouse genome enriched in CPE-containing genes are also enriched in *Drosophila*, suggesting a high level of conservation of CPE function between the two species.

### Cpeb1 promotes regeneration following neuronal injury

Our data suggest that CPE-enriched transcripts are temporarily protected from degradation after injury. Accordingly, we found that whereas the *cpeb1* transcript already decreases at 9 h following injury, the decrease in the protein level is delayed, starting at 24 h, as assessed by RT-PCR and WB of whole spinal tissues in naïve and at different time points following SCI (Figures S8A,B). Due to the lack of a high quality antibody for immunohistochemical staining of endogenous Cpeb1 in spinal cord tissue, we performed RNA FISH instead in combination with B3-tubulin staining (Figure S8C). Cpeb1 RNA was mainly found in B3-tubulin positive cells with a morphology characteristic of motor neurons. Altogether, these data indicate that Cpeb1 localizes to neurons, and while its transcript tend to decrease immediately following spinal cord injury, the decrease in protein levels is delayed to later time points, which may explain the observed protection of CPE-transcripts at 9 h following SCI.

**Figure 5:**
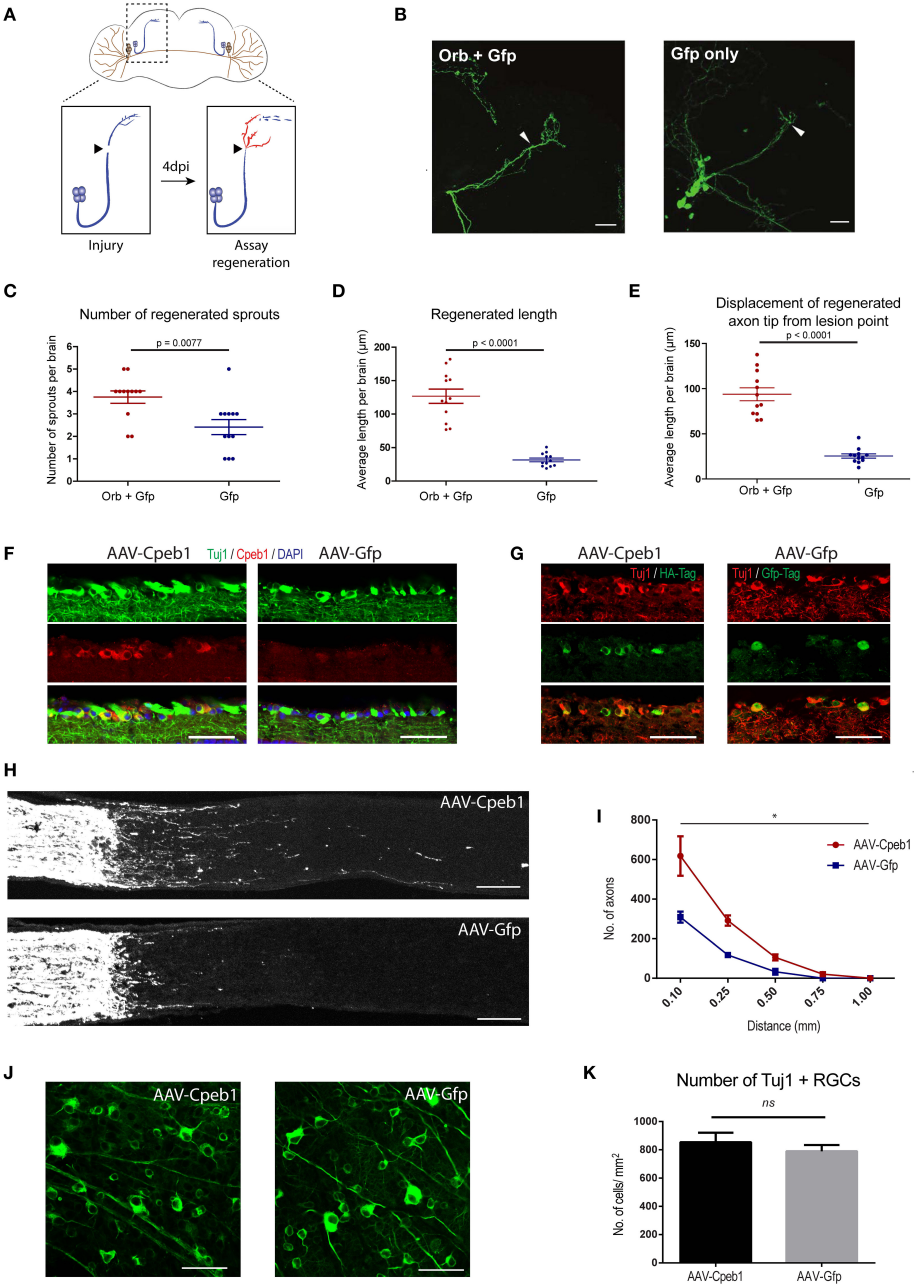
Cpeb1 overexpression promotes axonal regeneration in the adult mouse and *Drosophila* CNS. **(A–E)** Over-expression of Orb (Cpeb1 homolog) enhances axonal regeneration in *Drosophila* sLNv neurons 4 days after axotomy. **(A)** Experimental scheme. **(B)** Representative images. Arrowheads indicate lesion points. Scale bars: 30 μm. **(C–E)** Quantification of number, length, and displacement from lesion point of regenerated axon sprouts. Each point represents one brain slice from one fly. *n* = 13 (Orb+Gfp) or 12 (Gfp only) flies. Error bars: mean ± S.E.M. **(F–K)** AAV-driven over-expression of Cpeb1 enhances axonal regeneration following optic nerve crush injury without affecting RGC survival. **(F)** Sections of retinas from AAV-Cpeb1 or AAV-Gfp injected mice without optic nerve crush injury. Retinas were stained with Tuj1 (Green) and Cpeb1 (Red) antibodies. Scale bars: 50 μm. **(G)** Sections of retinas showing double staining for Tuj1 (Red) and HA-Tag (Green) from AAV-Cpeb1 injected mice and double staining for Tuj1 (Red) and Gfp-Tag (Green) from AAV-GFP injected mice at 2 weeks after crush injury. Scale bars: 50 μm. **(H)** Sections of optic nerves with CTB-labeled axons from WT mice injected with either AAV-Cpeb1 or AAV-Gfp at 2 weeks after optic nerve crush injury. Scale bars: 100 μm. **(I)** Quantification of regenerating axons at different distances distal to the lesion sites. *n* = 6 in each group. Statistical comparison between the two groups was performed with an exponential decrease model (^*^p = 0.0005 for difference in starting number of axons, *p* = 0.0759 for difference in rate of decrease across distance). **(J)** Whole-mount retinas from AAV-Cpeb1 or AAV-Gfp injected mice at 2 weeks after crush. Retinas were stained with Tuj1 (Green). Scale bars: 50 μm. **(K)** Quantification of the survival RGCs in retinas at 2 weeks after crush. *n* = 6 in each group.

To address the neuronal specific role of Cpeb1 in axonal regeneration, and specifically whether the failure to up-regulate Cpeb1 might in part explain the transient and abortive nature of the regenerative response to injury, we overexpressed the fly homolog of Cpeb1, Orb, exclusively in the sLNvs. Fly brains were dissected and kept in culture as described (Ayaz et al., 2008; Koch and Hassan, 2012; Koch et al., 2018). sLNvs were mechanically injured and the regenerative response was assessed after 4 days. 13 and 12 brains were analyzed for Orb+Gfp and Gfp only control respectively (Figures 5A,B). It was observed that the number of regenerated sprouts increased from a mean of 2.42 ± 0.336 (SEM) per brain in controls to 3.75 ± 0.279 (SEM) with the overexpression of Orb (Figure 5C). In addition, regenerated length was increased from 31.61 μm ± 2.87 (SEM) per brain in controls to 126.74 μm ± 10.6 (SEM) with Orb overexpression (Figure 5D). Likewise, displacement from the lesion point, which is the distance between the lesion point and the axon tip and accounts for proper pathfinding, also increased from 25.42 μm ± 2.39 (±SEM) per brain in controls to 93.76 μm ± 7.17 (SEM) with Orb overexpression. (Figure 5E).

To investigate whether this effect is conserved in mammals too, we turned to a mouse model of optic crush injury that allows overexpression of Cpeb1 in mouse retinal ganglion cells (RGCs). RGCs were infected with adeno-associated viral (AAV) vectors expressing Cpeb1-HA or Gfp under the neuron-specific human synapsin (hSyn) promoter. Thereafter, regeneration of RGC axons was assessed 2 weeks following a crush injury of the optic nerve. Six optic nerves from six animals with Cpeb1 overexpression and six control optic nerves with GFP virus infection were analyzed 2 weeks after injury. Staining of RGCs indicated a similar infection efficiency for AAV-Cpeb1-HA (79%) and the control AAV-Gfp (70%), and overexpression of Cpeb1 was verified by staining (Figures 5F,G). Regeneration was quantified as the number of regenerating axons reaching different distances from the lesion point. Importantly, as in *Drosophila*, over-expression of Cpeb1 in RGCs significantly increased the number and lengths of regenerating axons (Figures 5H,I). AAV-Cpeb1 infected RGCs start with approximately double the number of axons than AAV-Gfp infected ones at 0.1 mm past the lesion point (*p* = 0.0005). While the number of axons from both groups decrease across distance, this ratio is nearly maintained (test for difference in rate of decrease *p* = 0.0759), meaning that about twice the number of axons crosses distances up to 1.0 mm in the AAV-Cpeb1 infected RGCs than the control infected ones (Figure 5I). The number of survived RGCs in the retinas remained constant, indicating that the regenerative effect is not due to reduced cell death after injury (Figures 5J,K). To test whether knockout of Cpeb1 produces an opposite effect, we knocked out Cpeb1 in primary cultures of mouse cortical neurons. Efficient knockout is triggered via AAV-Cre mediated deletion of exon 4 (Figure S9A), which causes a frameshift that affects the activation and RNA recognition domains of Cpeb1. Neurons were cultured in a transwell chamber which specifically allows neurites to grow on the underside of the chamber. Scraping the lower side of the transwell mimicked a transection-injury. Thereafter, regenerating neurites on the underside were traced after 24 h (Figure S9B). Notably, knockout of Cpeb1 reduced the number of regenerating neurites from a mean of 447.41 ± 101.6 (SEM) to 167 ± 41.8 (SEM) per chamber (Figure S9C). The length of regenerated neurites was also reduced, albeit mildly, from 60.1 μm ± 2.14 (SEM) to 55.39 μm ± 1.57 (SEM) per chamber (Figure S9D). Together, these data support the notion that Cpeb1 is an enhancer of regeneration, and that this function is conserved between mice and *Drosophila*.

## Discussion

We profiled the responses to spinal cord injury both at transcriptome and translatome level, and find them to be highly uncoupled. A screening of factors showing this uncoupled behavior in *Drosophila* sLNvs revealed that 50% of the transcripts being prioritized for translation despite exhibiting reduced levels following spinal injury modulated axonal growth of developing neurons in the fly. Further, *in silico* analysis of these uncoupled-transcript led to identification of the CPE motif as highly conserved across species in functions including CNS-development. To influence expression of CPE-containing transcripts we overexpressed Cpeb1 in injured *Drosophila* and mammalian neurons. CPEB1 emerged as a conserved necessary and sufficient activator of neuronal regeneration. The current approach also illustrates the feasibility of uncovering novel functions of RNA-regulatory proteins by combining studies in mammalian CNS with screenings in model organisms, such as *Drosophila*.

The substantial uncoupling between transcription and translation in the injured spinal cord highlights the importance of post-transcriptional gene regulation in axon regeneration. The finding that the majority of genes are decreased in polysome-bound RNA fraction agrees with previous observations that stress conditions trigger a general shut down of translation to maximize cell survival (Park et al., 2008; Yamasaki and Anderson, 2008). The use of the uncoupled response in RNA regulation as a selection criteria proved to be more efficient at discovering novel factors influencing axonal growth than selection based on prior knowledge on their role in neural and neurite development (Koch et al., 2018). In addition, the fact that Cpeb1 has a positive role in axonal regeneration and the similar enrichment of CPE in the genomes of both mouse and *Drosophila*, indicates that the role of Cpeb1 is conserved across species.

These findings agree well with the increasing number of studies that report the role of axonal translation of specific mRNAs for axonal regeneration (Hanz et al., 2003; Perlson et al., 2005; Yudin et al., 2008; Yan et al., 2009; Perry et al., 2012). In order for localized translation to occur in axons, both mRNA, as well as translation machinery, such as ribosomes and translation factors (e.g., elF4E), need to be present. In the case of mRNAs, it was found that injury triggers substantial changes in the axonal mRNA repertoire in cortical neuron cultures (Taylor et al., 2009). In an *in vivo* setting, growth cone-associated mRNAs Gap-43, Nrn1, and ActB are increased in crush-injured regenerating sciatic nerve axons compared to naïve ones (Kalinski et al., 2015). The same study also reported that the presence of ribosome components and activated translation factors in sciatic nerve axons is induced by injury. A preconditioning injury that activates regeneration of secondarily injured DRG axons increases the rate of incorporation of radioactively labeled amino acids, and selective local axonal application of the translation inhibitor cycloheximide severely reduces regenerative response (Verma et al., 2005). Together, this confirms that axonal translation occurs upon injury. Remarkably, the levels of ribosome components, translation factors and growth cone-associated mRNAs in regenerated transected spinal cord axons were comparable to those of crush-injured regenerating sciatic axons. This suggests that increasing axonal protein synthesis of specific mRNAs may just be key to overcome the lack of regeneration in the CNS (Kalinski et al., 2015).

While we identified Cpeb1 as an enhancer of axonal regeneration and CPE to be associated with expression changes upon spinal cord injury, it is surprising that this occurs in total RNA and not polysome-bound RNAs, since Cpeb1 is best known for its role in cytoplasmic polyadenylation and translation control. However, Cpeb1 is also associated with a myriad of functions in post-transcriptional regulation, including alternative polyadenylation, RNA transport, storage, and degradation. Cpeb1 has been shown to transport CPE-containing mRNAs into dendrites in rat hippocampal neurons, in particular Map2, as ribonucleoprotein (RNP) and in a microtubule-dependent manner (Huang et al., 2003). In addition, Cpeb1 is present in stress granules and dcp1 bodies, which are subcellular structures for mRNA storage and degradation (Anderson and Kedersha, 2006; Eulalio et al., 2007). Overexpression of Cpeb1 increases the assembly of these structures and is dependent on its RNA binding domain (Wilczynska et al., 2005). Interestingly, this is not dependent on the phosphorylation site for activation of Cpeb1 during cytoplasmic polyadenylation. Similarly, deletion of the phosphorylation site does not alter the distribution of Cpeb1-containing foci within the dendrites and synapses of Xenopus optic tectal neurons, suggesting that the function of Cpeb1 to transport and target mRNAs to RNP complexes is independent of its role in cytoplasmic polyadenylation (Bestman and Cline, 2009). Foci containing inactive Cpeb1 mutants located near synapses do, however, show higher intensities than those containing wild-type Cpeb1, suggesting that inability to activate translation may trap Cpeb1 and its target mRNA within the RNP complex (Bestman and Cline, 2009). A possible scenario linking these observations is that inactive Cpeb1 forms RNP complexes together with the bound mRNA, leading to simultaneous repression of mRNA translation and protection from degradation while guiding them to the final location. Another scenario involves regulation of alternative polyadenylation by Cpeb1, thereby recruiting splicing factors to different polyadenylation sites and generating transcripts of varying 3′UTR lengths (Bava et al., 2013). This process affects the cis-elements present in the 3′UTR of the transcript (e.g., other RNA binding motifs or miRNA binding sites), which in turn affects localization, stability and translation efficiency of the transcript (Mayr and Bartel, 2009; Lianoglou et al., 2013). It is evident that from the many literature that Cpeb1 is capable of many different functions which may be context dependent. Indeed, one study even casted in doubt the fidelity of the relationship between Cpeb1 and CPE, where they found that there are proteins other than Cpeb1 that binds and act on CPE to regulate axon growth in Xenopus retinal axons (Lin et al., 2009). The obvious suspects are the other members of the Cpeb family which include Cpeb2, Cpeb3, and Cpeb4 that share structural similarity. At first glance this is unlikely, as Cpeb1 is the most distant member of the family, and in particular, it shares little similarity in its RNA binding domain with the other family members and they were shown to interact with different RNA sequences (Huang et al., 2006; Wang and Cooper, 2010). However, it was later shown that Cpeb4 could take over the function of Cpeb1 in meiosis (Igea and Méndez, 2010). These studies show that there is a need to carefully investigate the precise role of RNA binding proteins and their bind motifs in each individual context.

In summary, starting with a global approach, our study reveals the role of wide-spread post-transcriptional regulation in the early injury response in the spinal cord. While translation of mRNAs related to CNS development appears to be prioritized in the acute phase after injury, limitation in their transcript availability likely leads to the eventual failure in regeneration. By focusing on genes that exhibit uncoupling behavior between transcript availability and translation, we identified a number of genes that modulate outgrowth of axons during development, demonstrating that this as a viable method to identify neuronal intrinsic regulators of regeneration. We have also found the association of 3′UTR motifs with CNS injury response, and identified Cpeb1 as a modulator of axon regrowth, possibly by increasing the availability of development-related transcripts.

## Author contributions

WL performed experiments and wrote the manuscript. AM performed bioinformatics analysis and wrote the manuscript. MKo and MZ performed the *Drosophila* experiments. MG performed bioinformatics analysis. SKl, SKu, SL, CS, and RP performed experiments. MKe and MS helped establish the sucrose gradient fractionation. ES and DG generated the AAV vectors. RM and CM provided the transgenic mouse line, antibodies, and critical discussion of the data. CY and NL performed the optic nerve crush injury experiments supervised by KL who also wrote the manuscript. BH supervised the *Drosophila* part of the study and wrote the manuscript. AM-V designed and supervised the study and wrote the manuscript.

### Conflict of interest statement

The authors declare that the research was conducted in the absence of any commercial or financial relationships that could be construed as a potential conflict of interest.

## Supplementary material

Figure S1 Correlation and validation of microarray. **(A)** Correlation plot of normalized arrays (log2[normalized intensities]) for total and polysome-bound RNA fractions. **(B)** Comparison of expression changes of selected genes derived from microarray or qPCR. **(C)** Expression patterns of cell-type-specific genes are similar in both, conditions “naïve” and “injured.” Colors represent normalized intensities of microarray probes mapped to marker genes for motor neurones, other neurones, oligodendrocytes, microglia, precursors, and blood cells. The expression patterns between the conditions “naïve” and “injured” of these markers show a Pearsons' correlation of 0.97 for total RNA and 0.99 for RNA bound to polysomes.

Figure S2 **(A–F)** Representative images from developmental axonal growth screening for Orb (homolog of Cpeb1), Mask (homolog of Ankhd1), Smr (homolog of Ncor1), and Brat (homolog of Trim). Boxes highlight the developing axon tip. Arrowhead: dorsally projecting axons. Arrow: cell bodies. Scale bars: 50 μm.

Figure S3 Association of PBE, MBE, and Hex with expression changes upon SCI. Density curves of expression changes in **(A)** total and **(B)** polysome-bound RNA upon SCI of transcripts separated by the presence of PBE, MBE, and Hex in the 3′ UTR. Ticks on top and below the plots represent values of log2 (fold change) of individual transcripts. Distributions were compared with Kolmogorov-Smirnov test.

Figure S4 Association of PBE, MBE, and Hex with expression changes in transcripts from axon and CNS development GO categories upon SCI. Density curves of fold changes in total **(A)** and polysome-bound **(B)** RNA fractions of genes associated with GO categories of axon and CNS development, separated by those that contains PBE, MBE, or Hex and those that do not. There are no transcripts that do not contain Hex under axon development GO category. Ticks on top and below the plots represent values of log2 (fold change) of individual transcripts. Distributions were compared with Kolmogorov-Smirnov test.

Figure S5 Association of AREs with higher expression changes upon SCI and with CPE. **(A,B)** Density curves showing distribution of expression changes in total and polysome-bound RNA upon SCI of transcripts containing AREs in the 3′ UTR. **(C)** Number of transcripts harboring CPE and AREs in the whole transcriptome. **(D)** Density curves showing the distribution of expression changes of transcripts containing both AREs and CPE in the 3′UTR and those that do not in total and polysome-bound RNA upon SCI. Ticks on top and below the plots represent values of log2 (fold change) of individual transcripts. Distributions were compared with Kolmogorov-Smirnov test.

Figure S6 Association of AREs with PBE, MBE, and Hex and higher expression changes upon SCI. **(A–C)** Number of transcripts harboring AREs and PBE, MBE, or Hex in the whole transcriptome. **(D–F)** Density curves showing the distribution of expression changes of transcripts containing both AREs and PBE, MBE, or Hex in the 3'UTR and those that do not in total and polysome-bound RNA upon SCI. Ticks on top and below the plots represent values of log2 (fold change) of individual transcripts. Distributions were compared with Kolmogorov-Smirnov test.

Figure S7 Control analysis for motif analysis. Substituting the motif analysis with **(A)** the same motifs but in the 5′ UTR and **(B)** random motifs shows no association with expression changes.

Figure S8 Expression of Cpeb1 in spinal cord tissue. **(A)** Western blotting of Cpeb1 of naïve and injured spinal cords. Cell lysates of HT22 cells transiently over-expressing Cpeb1 were used as positive control. **(B)** qPCR of naïve and injured spinal cords for Cpeb1. (1-way ANOVA *p* = 0.9622). Error bars: mean± S.E.M. **(C)** Combined FISH and immunohistochemical staining for Cpeb1 RNA and B3-tubulin protein in naïve mouse spinal cord. Note that Cpeb1 transcript expression is restricted to neurons. Scale bars: 20 μm.

Figure S9 Deletion of Cpeb1 reduces neurite regeneration *in vitro*. **(A)** Efficient deletion of Cpeb1 by AAV-delivered Cre confirmed by PCR. Expected sizes: 884 bp (without deletion), 186 bp (with deletion). **(B)** Cortical neurons infected with AAV-Cre were seeded on transwell chambers, which exclusively allow neurite growth on the lower side of the membrane. Representative images of neurites skeletonized from image processing. **(C,D)** Quantification of regenerating neurites 24 h after injury. Each data point represents one culture chamber. A total of 29 culture chambers prepared from four mice were used per group. Cpeb1 ko: Cpeb1^flox/flox^ + AAV; wt: wild-type + AAV. Scale bars: 100 μm. Error bars: mean ± S.E.M.

Table S1 Expression fold change in the total and polysome-bound RNA fractions of all probesets in the microarray following spinal cord injury.

Table S2 Expression values in the microarray of probesets mapping to cell-type-specific marker genes.

Table S3 Gene Ontology enrichment analysis of up-regulated genes as compared to all differentially regulated genes from the total and polysome-bound RNA fractions in microarray. Over- and under-representation of up-regulated genes corresponds to an enrichment of up-regulated and down-regulated genes respectively in that category.

Table S4 List of fly lines used in the outgrowth screening.

Table S5 List of sequences used in motif analysis.

Table S6 Gene Ontology enrichment analysis of CPE-containing genes in mouse and fly genomes, showing GO categories with over- and under-representation of genes with transcripts containing CPE.

## References

[B1] Anderson P. Kedersha N. (2006). RNA granules. J. Cell Biol. 172, 803–808. 10.1083/jcb.200512082 16520386 PMC2063724

[B2] Ashburner M. Ball C. A. Blake J. A. Botstein D. Butler H. Cherry J. M.. (2011). The gene ontology consortium. Gene ontology: tool for the unification of biology. Nat. Genet. 25, 25–29. 10.1038/75556 10802651 PMC3037419

[B3] Ayaz D. Leyssen M. Koch M. Yan J. Srahna M. Sheeba V.. (2008). Axonal injury and regeneration in the adult brain of *Drosophila*. J. Neurosci. 28, 6010–6021. 10.1523/JNEUROSCI.0101-08.2008 18524906 PMC2693324

[B4] Bareyre F. M. Kerschensteiner M. Raineteau O. Mettenleiter T. C. Weinmann O. Schwab M. E. (2004). The injured spinal cord spontaneously forms a new intraspinal circuit in adult rats. Nat. Neurosci. 7, 269–277. 10.1038/nn1195 14966523

[B5] Bava F.-A. Eliscovich C. Ferreira P. G. Miñana B. Ben-Dov C. Guigó R.. (2013). CPEB1 coordinates alternative 3′-UTR formation with translational regulation. Nature 495, 121–125. 10.1038/nature11901 23434754

[B6] Bernstein D. R. Stelzner D. J. (1983). Plasticity of the corticospinal tract following midthoracic spinal injury in the postnatal rat. J. Comp. Neurol. 221, 382–400. 10.1002/cne.902210403 6662981

[B7] Bestman J. E. Cline H. T. (2009). The relationship between dendritic branch dynamics and CPEB-labeled RNP granules captured *in vivo*. Front. Neural Circuits 3:10. 10.3389/neuro.04.010.2009 19753328 PMC2742666

[B8] Blichenberg A. Schwanke B. Rehbein M. Garner C. C. Richter D. Kindler S. (1999). Identification of a cis-acting dendritic targeting element in MAP2 mRNAs. J. Neurosci. 19, 8818–8829. 10516301 10.1523/JNEUROSCI.19-20-08818.1999 PMC6782761

[B9] Brand A. H. Perrimon N. (1993). Targeted gene expression as a means of altering cell fates and generating dominant phenotypes. Development 118, 401–415. 8223268 10.1242/dev.118.2.401

[B10] Bregman B. S. Kunkel-Bagden E. McAtee M. O'Neill A. (1989). Extension of the critical period for developmental plasticity of the corticospinal pathway. J. Comp. Neurol. 282, 355–370. 10.1002/cne.902820304 2715387

[B11] Cajal S. R. DeFelipe J. Jones E. G. (1991). Cajal's Degeneration and Regeneration of the Nervous System. Oxford, UK: Oxford University Press.

[B12] Charlesworth A. Meijer H. A. de Moor C. H. (2013). Specificity factors in cytoplasmic polyadenylation. Wiley Interdiscipl. Rev. 4, 437–461. 10.1002/wrna.1171 23776146 PMC3736149

[B13] Colgan D. F. Manley J. L. (1997). Mechanism and regulation of mRNA polyadenylation. Genes Dev. 11, 2755–2766. 10.1101/gad.11.21.2755 9353246

[B14] Côté M.-P. Amin A. A. Tom V. J. Houle J. D. (2011). Peripheral nerve grafts support regeneration after spinal cord injury. Neurotherapeutics 8, 294–303. 10.1007/s13311-011-0024-6 21360238 PMC3101823

[B15] Darnell J. C. Richter J. D. (2012). Cytoplasmic RNA-binding proteins and the control of complex brain function. Cold Spring Harb. Perspect. Biol. 4:a012344. 10.1101/cshperspect.a012344 22723494 PMC3405866

[B16] Davies S. J. Fitch M. T. Memberg S. P. Hall A. K. Raisman G. Silver J. (1997). Regeneration of adult axons in white matter tracts of the central nervous system. Nature 390, 680–683. 10.1038/37776 9414159

[B17] Demjen D. Klussmann S. Kleber S. Zuliani C. Stieltjes B. Metzger C.. (2004). Neutralization of CD95 ligand promotes regeneration and functional recovery after spinal cord injury. Nat. Med. 10, 389–395. 10.1038/nm1007 15004554

[B18] Donnelly C. J. Willis D. E. Xu M. Tep C. Jiang C. Yoo S.. (2011). Limited availability of ZBP1 restricts axonal mRNA localization and nerve regeneration capacity. EMBO J. 30, 4665–4677. 10.1038/emboj.2011.347 21964071 PMC3243598

[B19] Eulalio A. Behm-Ansmant I. Izaurralde E. (2007). P bodies: at the crossroads of post-transcriptional pathways. Nat. Rev. Mol. Cell Biol. 8, 9–22. 10.1038/nrm2080 17183357

[B20] Falcon S. Gentleman R. (2007). Using GOstats to test gene lists for GO term association. Bioinformatics 23, 257–258. 10.1093/bioinformatics/btl567 17098774

[B21] Filbin M. T. (2006). Recapitulate development to promote axonal regeneration: good or bad approach? Philos. Trans. R. Soc. B Biol. Sci. 361, 1565–1574. 10.1098/rstb.2006.1885 PMC1664663 16939975

[B22] Fontana X. Hristova M. Da Costa C. Patodia S. Thei L. Makwana M.. (2012). c-Jun in Schwann cells promotes axonal regeneration and motoneuron survival via paracrine signaling. J. Cell Biol. 198, 127–141. 10.1083/jcb.201205025 22753894 PMC3392945

[B23] Gadani S. P. Walsh J. T. Lukens J. R. Kipnis J. (2015). Dealing with danger in the CNS: the response of the immune system to Injury. Neuron 87, 47–62. 10.1016/j.neuron.2015.05.019 26139369 PMC4491143

[B24] Gingerich T. J. Feige J. J. LaMarre J. (2004). AU-rich elements and the control of gene expression through regulated mRNA stability. Anim. Health Res. Rev. 5, 49–63. 10.1079/AHR200460 15460540

[B25] Hadziselimovic N. Vukojevic V. Peter F. Milnik A. Fastenrath M. Fenyves B. G.. (2014). Forgetting is regulated via Musashi-mediated translational control of the Arp2/3 complex. Cell 156, 1153–1166. 10.1016/j.cell.2014.01.054 24630719

[B26] Hanz S. Perlson E. Willis D. Zheng J. Q. Massarwa R. Huerta J. J.. (2003). Axoplasmic importins enable retrograde injury signaling in lesioned nerve. Neuron 40, 1095–1104. 10.1016/S0896-6273(03)00770-0 14687545

[B27] Hausmann O. N. (2003). Post-traumatic inflammation following spinal cord injury. Spinal Cord 41, 369–378. 10.1038/sj.sc.3101483 12815368

[B28] Holt C. E. Schuman E. M. (2013). The central dogma decentralized: new perspectives on RNA function and local translation in neurons. Neuron 80, 648–657. 10.1016/j.neuron.2013.10.036 24183017 PMC3820025

[B29] Hoopfer E. D. McLaughlin T. Watts R. J. Schuldiner O. O'Leary D. D. M. Luo L. (2006). Wlds protection distinguishes axon degeneration following injury from naturally occurring developmental pruning. Neuron 50, 883–895. 10.1016/j.neuron.2006.05.013 16772170

[B30] Huang Y. S. Carson J. H. Barbarese E. Richter J. D. (2003). Facilitation of dendritic mRNA transport by CPEB. Genes Dev. 17, 638–653. 10.1101/gad.1053003 12629046 PMC196011

[B31] Huang Y. S. Kan M. C. Lin C. L. Richter J. D. (2006). CPEB3 and CPEB4 in neurons: analysis of RNA-binding specificity and translational control of AMPA receptor GluR2 mRNA. EMBO J. 25, 4865–4876. 10.1038/sj.emboj.7601322 17024188 PMC1618119

[B32] Huber W. von Heydebreck A. Sültmann H. Poustka A. Vingron M. (2002). Variance stabilization applied to microarray data calibration and to the quantification of differential expression. Bioinformatics 18, S96–S104. 10.1093/bioinformatics/18.suppl_1.S96 12169536

[B33] Igea A. Méndez R. (2010). Meiosis requires a translational positive loop where CPEB1 ensues its replacement by CPEB4. EMBO J. 29, 2182–2193. 10.1038/emboj.2010.111 20531391 PMC2905248

[B34] Ivshina M. Lasko P. Richter J. D. (2014). Cytoplasmic polyadenylation element binding proteins in development, health, and disease. Annu. Rev. Cell Dev. Biol. 30, 393–415. 10.1146/annurev-cellbio-101011-155831 25068488

[B35] Jung H. Yoon B. C. Holt C. E. (2012). Axonal mRNA localization and local protein synthesis in nervous system assembly, maintenance and repair. Nat. Rev. Neurosci. 13, 308–324. 10.1038/nrn3274 22498899 PMC3682205

[B36] Kalinski A. L. Sachdeva R. Gomes C. Lee S. J. Shah Z. Houle J. D.. (2015). mRNAs and protein synthetic machinery localize into regenerating spinal cord axons when they are provided a substrate that supports growth. J. Neurosci. 35, 10357–10370. 10.1523/JNEUROSCI.1249-15.2015 26180210 PMC4502271

[B37] Kaye J. A. Rose N. C. Goldsworthy B. Goga A. L'Etoile N. D. (2009). A 3'UTR pumilio-binding element directs translational activation in olfactory sensory neurons. Neuron 61, 57–70. 10.1016/j.neuron.2008.11.012 19146813 PMC4274156

[B38] Koch M. H. Hassan B. A. (2012). Out with the brain: *Drosophila* whole-brain explant culture, in The Making and Un-Making of Neuronal Circuits in *Drosophila*, Vol. 69, ed Hassan B. A. (Totowa, NJ: Humana Press), 261–268. 10.1007/978-1-61779-830-6_12

[B39a] Koch M. Nicolas M. Zschaetzsch M. de Geest N. Claeys A. Yan J. (2018). A Fat-Facets-Dscam1-JNK pathway enhances axonal growth in development and after injury. Front. Cell. Neurosci. 12:416 10.3389/fncel.2017.00416 PMC5809495 29472843

[B39] Lau A. G. Irier H. A. Gu J. Tian D. Ku L. Liu G.. (2010). Distinct 3'UTRs differentially regulate activity-dependent translation of brain-derived neurotrophic factor (BDNF). Proc. Natl. Acad. Sci. U.S.A. 107, 15945–15950. 10.1073/pnas.1002929107 20733072 PMC2936648

[B40] Lee D.-H. Lee J. K. (2013). Animal models of axon regeneration after spinal cord injury. Neurosci. Bull. 29, 436–444. 10.1007/s12264-013-1365-4 23893429 PMC3920733

[B41] Letellier E. Kumar S. Sancho-Martinez I. Krauth S. Funke-Kaiser A. Laudenklos S.. (2010). CD95-ligand on peripheral myeloid cells activates Syk kinase to trigger their recruitment to the inflammatory site. Immunity 32, 240–252. 10.1016/j.immuni.2010.01.011 20153221

[B42] Lianoglou S. Garg V. Yang J. L. Leslie C. S. Mayr C. (2013). Ubiquitously transcribed genes use alternative polyadenylation to achieve tissue-specific expression. Genes Dev. 27, 2380–2396. 10.1101/gad.229328.113 24145798 PMC3828523

[B43] Lin A. C. Tan C. L. Lin C. L. Strochlic L. Huang Y. S. Richter J. D.. (2009). Cytoplasmic polyadenylation and cytoplasmic polyadenylation element-dependent mRNA regulation are involved in Xenopus retinal axon development. Neural Dev. 4, 8–20. 10.1186/1749-8104-4-8 19254368 PMC2661069

[B44] Liu K. Tedeschi A. Park K. K. He Z. (2011). Neuronal intrinsic mechanisms of axon regeneration. Annu. Rev. Neurosci. 34, 131–152. 10.1146/annurev-neuro-061010-113723 21438684

[B45] Lou W. P.-K. Baser A. Klußmann S. Martin-Villalba A. (2014). *In vivo* interrogation of central nervous system translatome by polyribosome fractionation. J. Vis. Exp. 86:e51255 10.3791/51255 PMC4183125 24835574

[B46] MacDonald J. M. Beach M. G. Porpiglia E. Sheehan A. E. Watts R. J. Freeman M. R. (2006). The *Drosophila* cell corpse engulfment receptor draper mediates glial clearance of severed axons. Neuron 50, 869–881. 10.1016/j.neuron.2006.04.028 16772169

[B47] Mar F. M. Bonni A. Sousa M. M. (2014). Cell intrinsic control of axon regeneration. EMBO Rep. 15, 254–263. 10.1002/embr.201337723 24531721 PMC3989691

[B48] Mayr C. Bartel D. P. (2009). Widespread shortening of 3′UTRs by alternative cleavage and polyadenylation activates oncogenes in cancer cells. Cell 138, 673–684. 10.1016/j.cell.2009.06.016 19703394 PMC2819821

[B49] Moore M. J. (2005). From birth to death: the complex lives of eukaryotic mRNAs. Science 309, 1514–1518. 10.1126/science.1111443 16141059

[B50] Nakamura M. Okano H. Blendy J. A. Montell C. (1994). Musashi, a neural RNA-binding protein required for drosophila adult external sensory organ development. Neuron 13, 67–81. 10.1016/0896-6273(94)90460-X 8043282

[B51] Park K. K. Liu K. Hu Y. Smith P. D. Wang C. Cai B.. (2008). Promoting axon regeneration in the adult CNS by modulation of the PTEN/mTOR pathway. Science 322, 963–966. 10.1126/science.1161566 18988856 PMC2652400

[B52] Perlson E. Hanz S. Ben-Yaakov K. Segal-Ruder Y. Seger R. Fainzilber M. (2005). Vimentin-dependent spatial translocation of an activated MAP kinase in injured nerve. Neuron 45, 715–726. 10.1016/j.neuron.2005.01.023 15748847

[B53] Perry R. B. Doron-Mandel E. Iavnilovitch E. Rishal I. Dagan S. Y. Tsoory M.. (2012). Subcellular knockout of importin β1 perturbs axonal retrograde signaling. Neuron 75, 294–305. 10.1016/j.neuron.2012.05.033 22841314 PMC3408616

[B54] Piqué M. López J. M. Foissac S. Guigó R. Méndez R. (2008). A combinatorial code for CPE-mediated translational control. Cell 132, 434–448. 10.1016/j.cell.2007.12.038 18267074

[B55] Schwanhäusser B. Busse D. Li N. Dittmar G. Schuchhardt J. Wolf J.. (2011). Global quantification of mammalian gene expression control. Nature 473, 337–342. 10.1038/nature10098 21593866

[B56] Shannon P. Markiel A. Ozier O. Baliga N. S. Wang J. T. Ramage D.. (2003). Cytoscape: a software environment for integrated models of biomolecular interaction networks. Genome Res. 13, 2498–2504. 10.1101/gr.1239303 14597658 PMC403769

[B57] Si K. Choi Y.-B. White-Grindley E. Majumdar A. Kandel E. R. (2010). Aplysia CPEB can form prion-like multimers in sensory neurons that contribute to long-term facilitation. Cell 140, 421–435. 10.1016/j.cell.2010.01.008 20144764

[B58] Smyth G. K. (2004). Linear models and empirical bayes methods for assessing differential expression in microarray experiments. Stat. Appl. Genet. Mol. Biol. 3, 1–25. 10.2202/1544-6115.1027 16646809

[B59] Song Y. Ori-McKenney K. M. Zheng Y. Han C. Jan L. Y. Jan Y. N. (2012). Regeneration of *Drosophila* sensory neuron axons and dendrites is regulated by the Akt pathway involving *Pten* and microRNA *bantam*. Genes Dev. 26, 1612–1625. 10.1101/gad.193243.112 22759636 PMC3404388

[B60] Spasic M. Friedel C. C. Schott J. Kreth J. Leppek K. Hofmann S.. (2012). Genome-wide assessment of AU-rich elements by the AREScore algorithm. PLoS Genet. 8:e1002433. 10.1371/journal.pgen.1002433 22242014 PMC3252268

[B61] Steward O. Zheng B. Tessier-Lavigne M. Hofstadter M. Sharp K. Yee K. M. (2008). Regenerative growth of corticospinal tract axons via the ventral column after spinal cord injury in mice. J. Neurosci. 28, 6836–6847. 10.1523/JNEUROSCI.5372-07.2008 18596159 PMC2745399

[B62] Stieltjes B. Klussmann S. Bock M. Umathum R. Mangalathu J. Letellier E.. (2006). Manganese-enhanced magnetic resonance imaging for *in vivo* assessment of damage and functional improvement following spinal cord injury in mice. Magn. Reson. Med. 55, 1124–1131. 10.1002/mrm.20888 16602070

[B63] Szostak E. Gebauer F. (2013). Translational control by 3'-UTR-binding proteins. Brief. Funct. Genomics 12, 58–65. 10.1093/bfgp/els056 23196851 PMC3548161

[B64] Taylor A. M. Berchtold N. C. Perreau V. M. Tu C. H. Li Jeon N. Cotman C. W. (2009). Axonal mRNA in uninjured and regenerating cortical mammalian axons. J. Neurosci. 29, 4697–4707. 10.1523/JNEUROSCI.6130-08.2009 19369540 PMC3632375

[B65] Tian B. Hu J. Zhang H. Lutz C. S. (2005). A large-scale analysis of mRNA polyadenylation of human and mouse genes. Nucleic Acids Res. 33, 201–212. 10.1093/nar/gki158 15647503 PMC546146

[B66] Trivedi A. Olivas A. D. Noble-Haeusslein L. J. (2006). Inflammation and spinal cord injury: infiltrating leukocytes as determinants of injury and repair processes. Clin. Neurosci. Res. 6, 283–292. 10.1016/j.cnr.2006.09.007 18059979 PMC1864937

[B67] Udagawa T. Swanger S. A. Takeuchi K. Kim J. H. Nalavadi V. Shin J.. (2012). Bidirectional control of mRNA translation and synaptic plasticity by the cytoplasmic polyadenylation complex. Mol. Cell 47, 1–14. 10.1016/j.molcel.2012.05.016 22727665 PMC3408552

[B68] Verma P. Chierzi S. Codd A. M. Campbell D. S. Meyer R. L. Holt C. E.. (2005). Axonal protein synthesis and degradation are necessary for efficient growth cone regeneration. J. Neurosci. 25, 331–342. 10.1523/JNEUROSCI.3073-04.2005 15647476 PMC3687202

[B69] Vessey J. P. Vaccani A. Xie Y. Dahm R. Karra D. Kiebler M. A.. (2006). Dendritic localization of the translational repressor Pumilio 2 and its contribution to dendritic stress granules. J. Neurosci. 26, 6496–6508. 10.1523/JNEUROSCI.0649-06.2006 16775137 PMC6674044

[B70] von Roretz C. Di Marco S. Mazroui R. Gallouzi I. E. (2011). Turnover of AU-rich-containing mRNAs during stress: a matter of survival. Wiley Interdiscip. Rev. 2, 336–347. 10.1002/wrna.55 21957021

[B71] Wang X.-P. Cooper N. G. F. (2010). Comparative *in silico* analyses of cpeb1-4 with functional predictions. Bioinform. Biol. Insights 4, 61–83. 10.4137/BBI.S5087 20838664 PMC2935813

[B72] Weill L. Belloc E. Bava F.-A. Méndez R. (2012). Translational control by changes in poly(A) tail length: recycling mRNAs. Nat. Struct. Mol. Biol. 19, 577–585. 10.1038/nsmb.2311 22664985

[B73] Wilczynska A. Aigueperse C. Kress M. Dautry F. Weil D. (2005). The translational regulator CPEB1 provides a link between dcp1 bodies and stress granules. J. Cell Sci. 118, 981–992. 10.1242/jcs.01692 15731006

[B74] Wu L. Wells D. Tay J. Mendis D. Abbott M. A. Barnitt A.. (1998). CPEB-mediated cytoplasmic polyadenylation and the regulation of experience-dependent translation of alpha-CaMKII mRNA at synapses. Neuron 21, 1129–1139. 10.1016/S0896-6273(00)80630-3 9856468

[B75] Xue F. Wu K. Wang T. Cheng Y. Jiang M. Ji J. (2016). Morphological and functional changes of the optic nerve following traumatic optic nerve injuries in rabbits. Biomed. Rep. 4, 188–192. 10.3892/br.2016.567 26893836 PMC4734054

[B76] Yamasaki S. Anderson P. (2008). Reprogramming mRNA translation during stress. Curr. Opin. Cell Biol. 20, 222–226. 10.1016/j.ceb.2008.01.013 18356035 PMC2841789

[B77] Yan D. Wu Z. Chisholm A. D. Jin Y. (2009). The DLK-1 kinase promotes mRNA stability and local translation in *C. elegans* synapses and axon regeneration. Cell 138, 1005–1018. 10.1016/j.cell.2009.06.023 19737525 PMC2772821

[B78] Ylera B. Ertürk A. Hellal F. Nadrigny F. Hurtado A. Tahirovic S.. (2009). Chronically CNS-injured adult sensory neurons gain regenerative competence upon a lesion of their peripheral axon. Curr. Biol. 19, 930–936. 10.1016/j.cub.2009.04.017 19409789

[B79] Young W. (2014). Spinal cord regeneration. Cell Transplant. 23, 573–611. 10.3727/096368914X678427 24816452

[B80] Yudin D. Hanz S. Yoo S. Iavnilovitch E. Willis D. Gradus T.. (2008). Localized regulation of axonal RanGTPase controls retrograde injury signaling in peripheral nerve. Neuron 59, 241–252 10.1016/j.neuron.2008.05.029 18667152 PMC2538677

