# Supplementary figures and images for: Regulation of Adult CNS Axonal Regeneration by the Post-transcriptional Regulator Cpeb1
Source: Front Mol Neurosci. 2018 Jan 12;10:445. doi: 10.3389/fnmol.2017.00445 (PMC5770975; doi:10.3389/fnmol.2017.00445)

A)

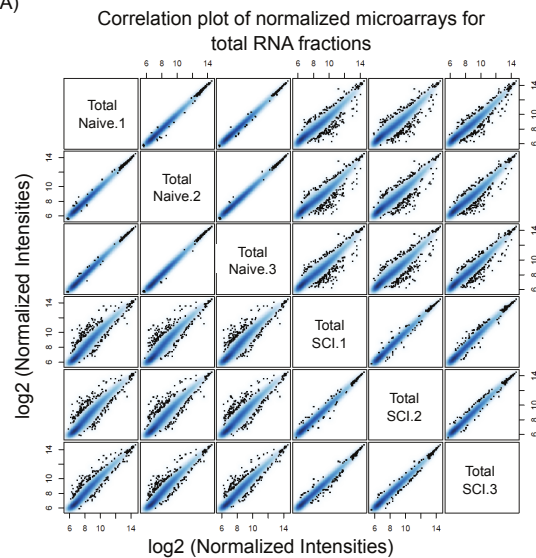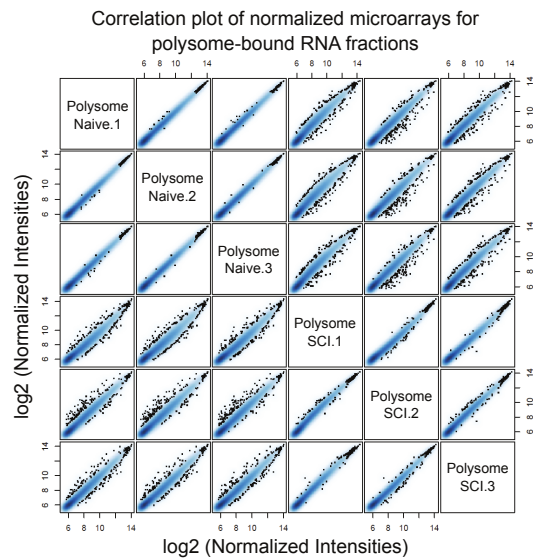

B)

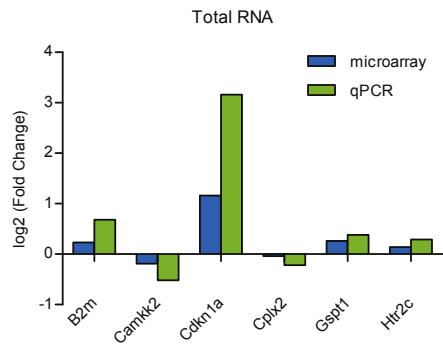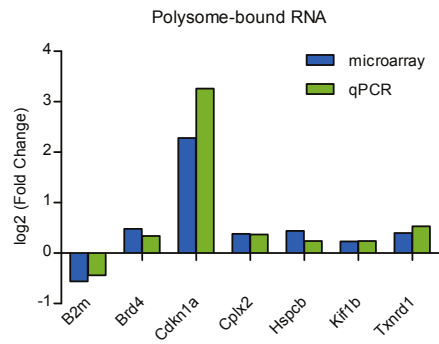

C)

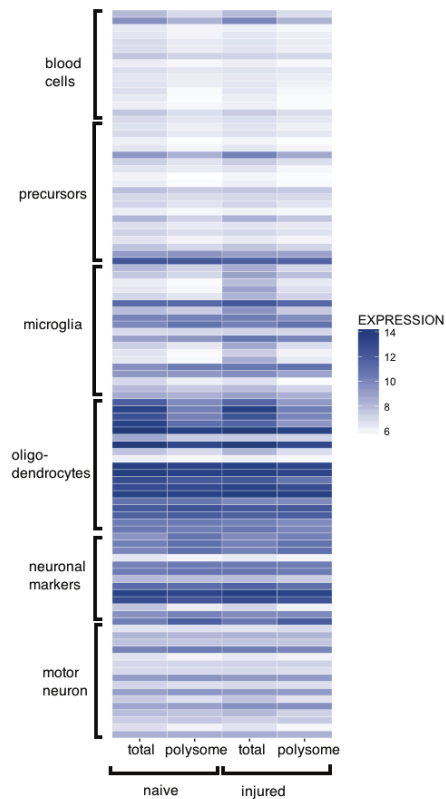

Supplement: Figure S1 — Correlation and validation of microarray. (A) Correlation plot of normalized arrays (log2[normalized intensities]) for total and polysome-bound RNA fractions. (B) Comparison of expression changes of selected genes derived from microarray or qPCR. (C) Expression patterns of cell-type-specific genes are similar in both, conditions “naïve” and “injured.” Colors represent normalized intensities of microarray probes mapped to marker genes for motor neurones, other neurones, oligodendrocytes, microglia, precursors, and blood cells. The expression patterns between the conditions “naïve” and “injured” of these markers show a Pearsons' correlation of 0.97 for total RNA and 0.99 for RNA bound to polysomes. [file Image1.PDF]

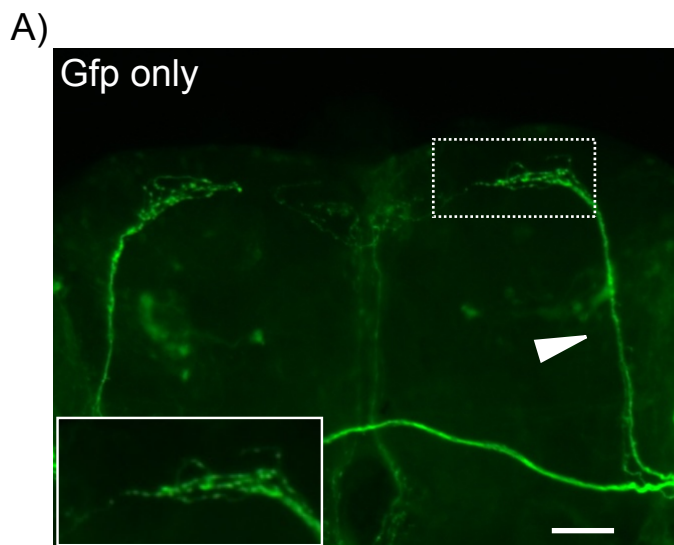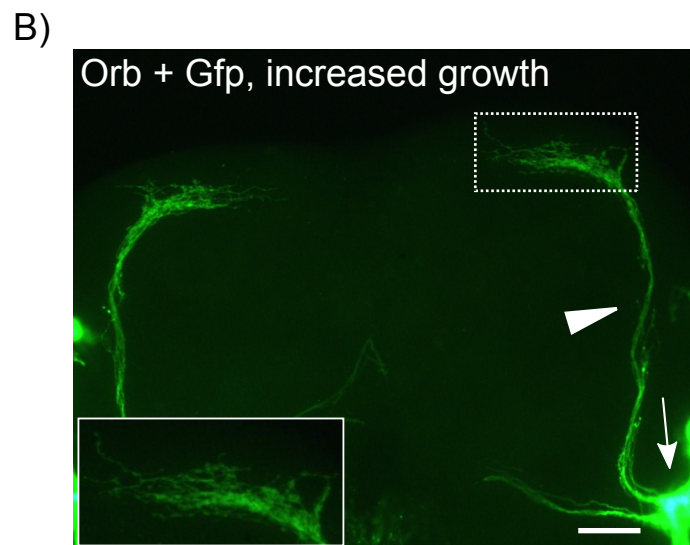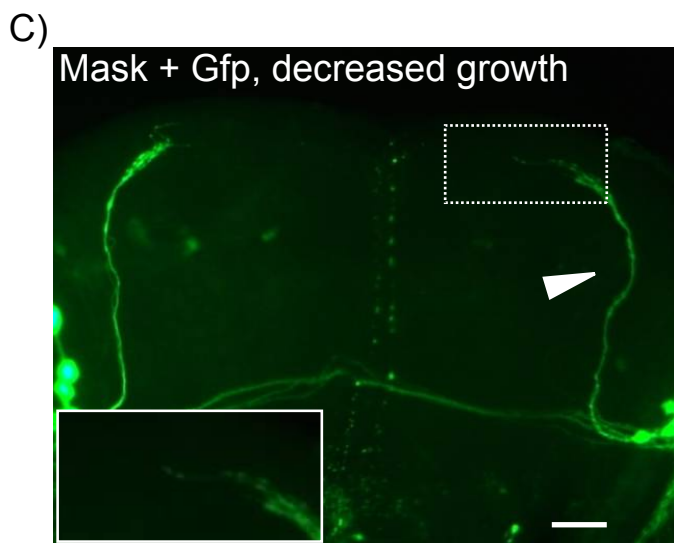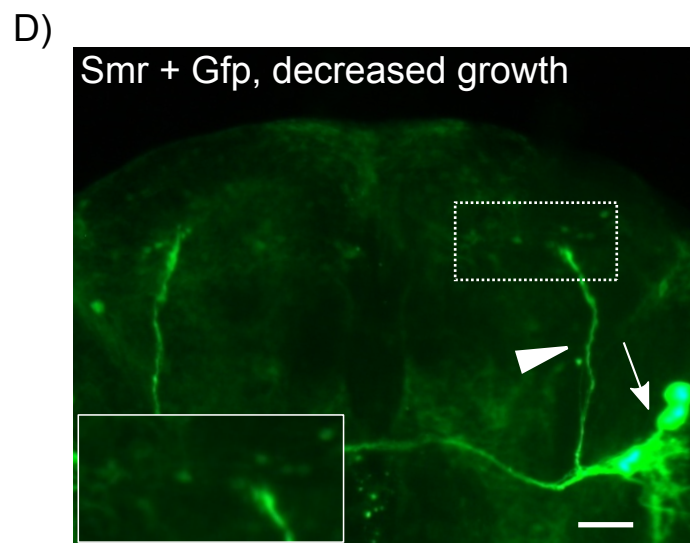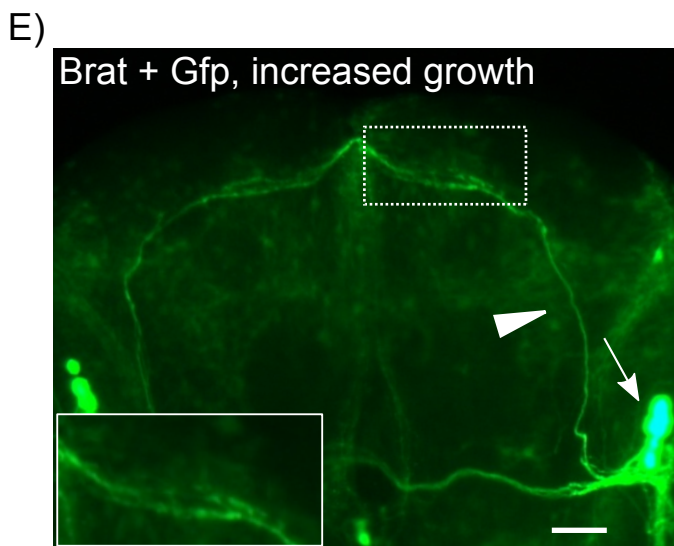

Supplement: Figure S2 — (A–F) Representative images from developmental axonal growth screening for Orb (homolog of Cpeb1), Mask (homolog of Ankhd1), Smr (homolog of Ncor1), and Brat (homolog of Trim). Boxes highlight the developing axon tip. Arrowhead: dorsally projecting axons. Arrow: cell bodies. Scale bars: 50 μm. [file Image2.PDF]

A)

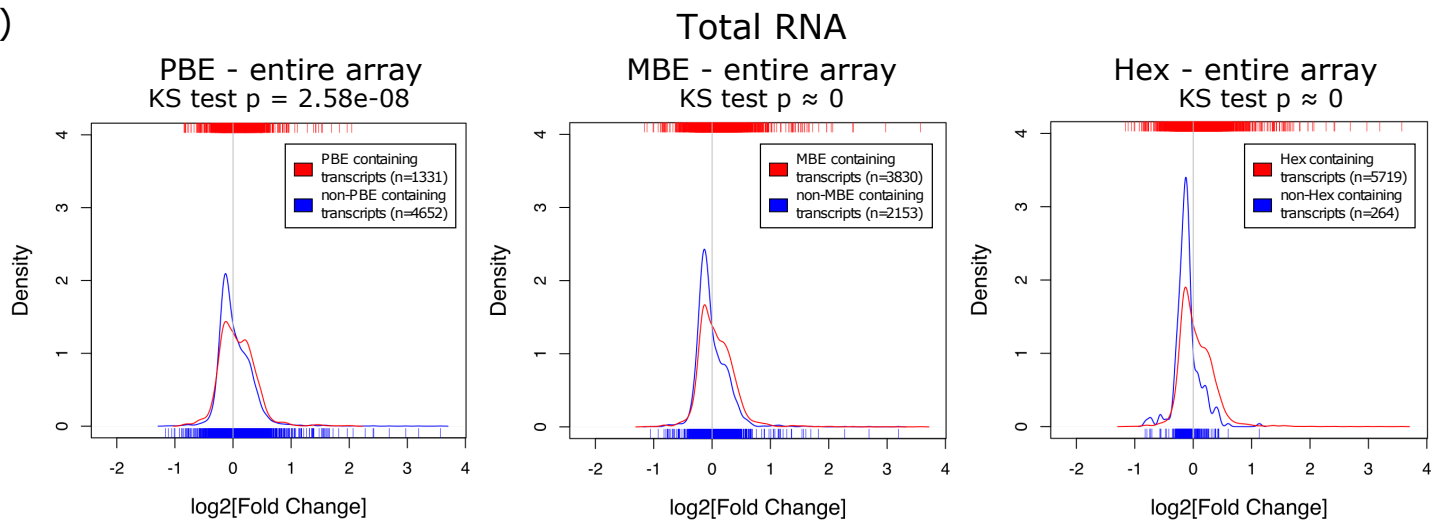

B)

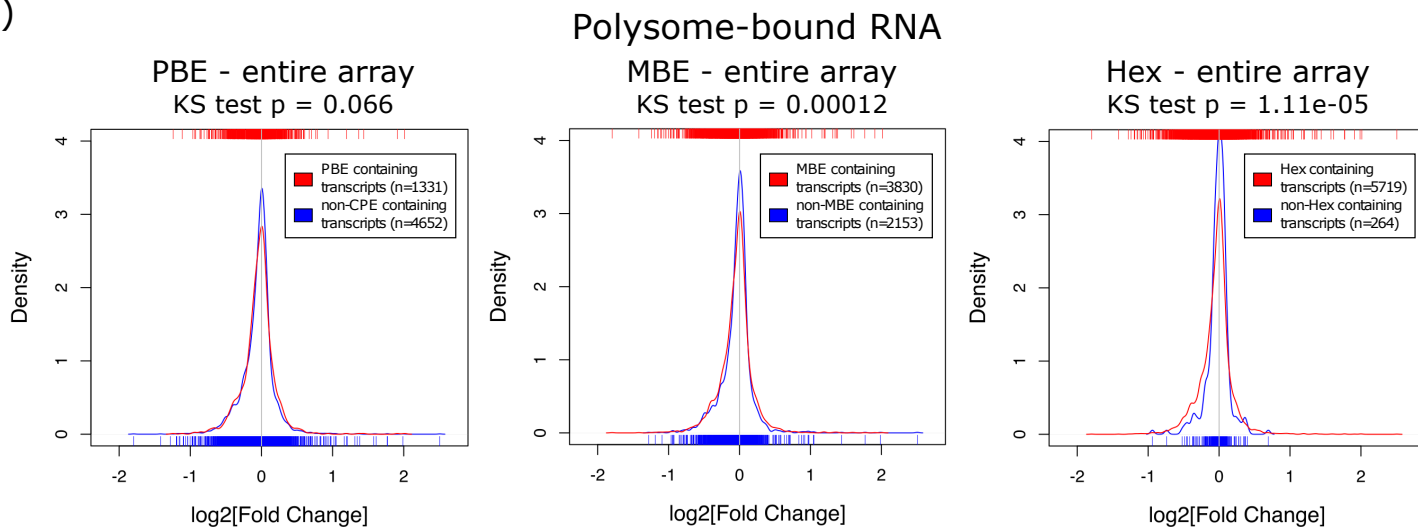

Supplement: Figure S3 — Association of PBE, MBE, and Hex with expression changes upon SCI. Density curves of expression changes in (A) total and (B) polysome-bound RNA upon SCI of transcripts separated by the presence of PBE, MBE, and Hex in the 3′ UTR. Ticks on top and below the plots represent values of log2 (fold change) of individual transcripts. Distributions were compared with Kolmogorov-Smirnov test. [file Image3.PDF]

A)

Total RNA

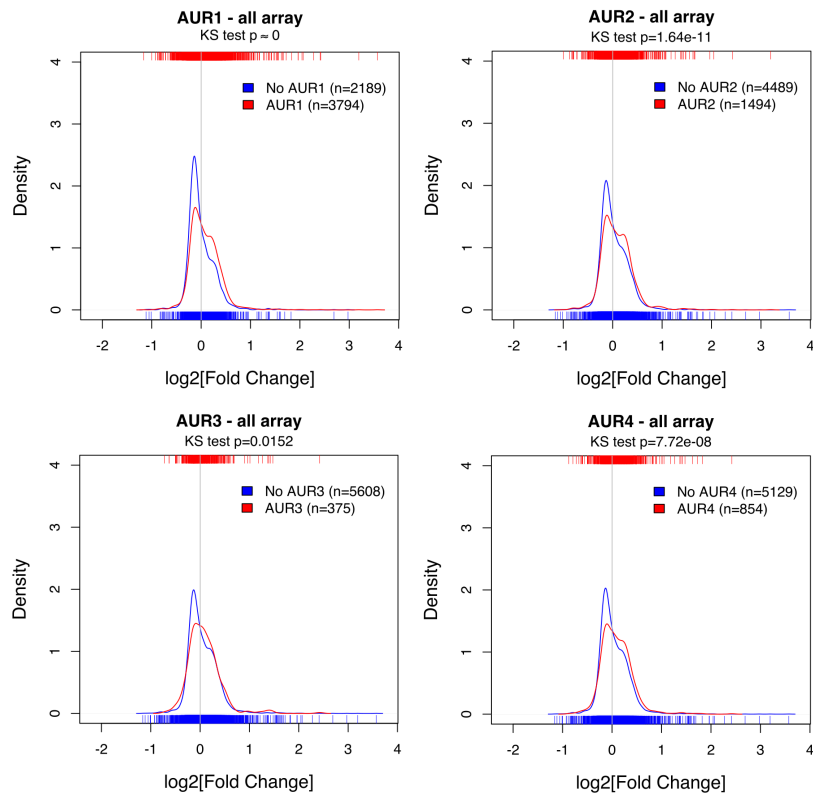

C)

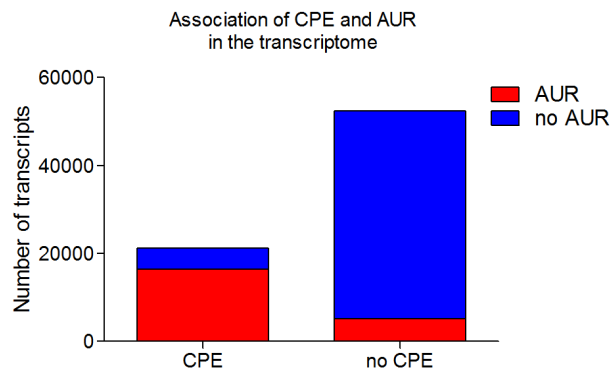

B)

Polysome-bound RNA

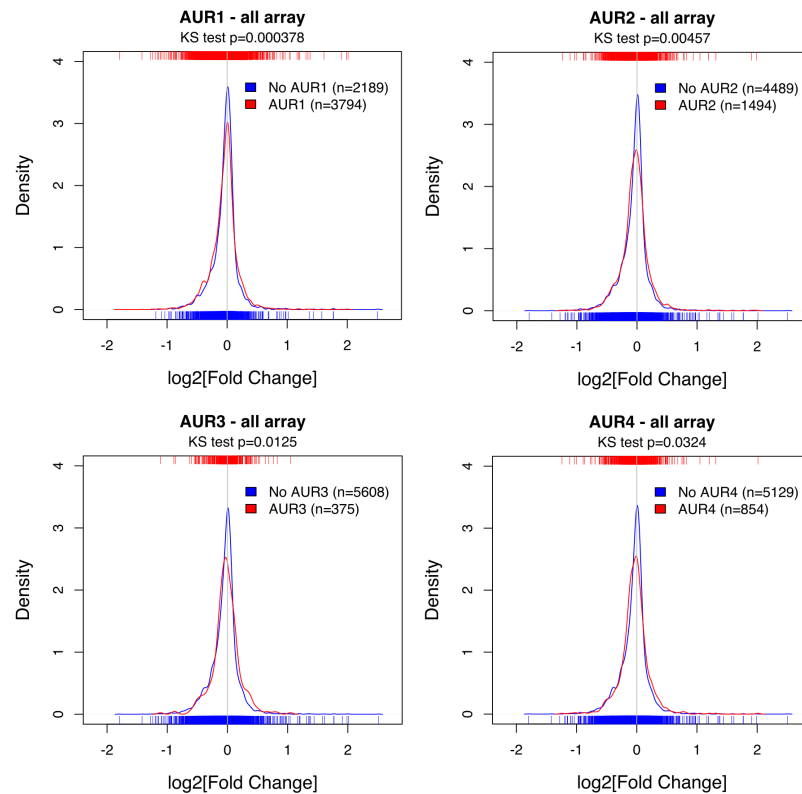

D)

Total RNA

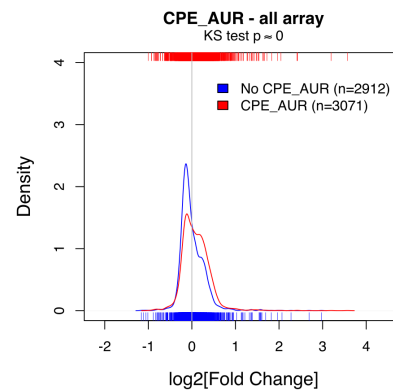

Polysome-bound RNA

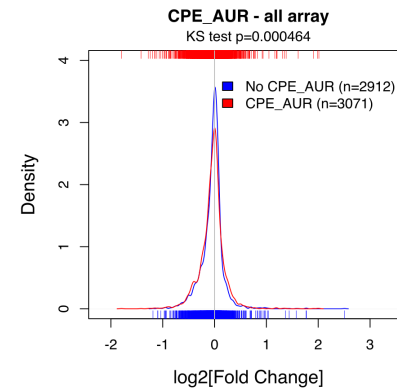

Supplement: Figure S5 — Association of AREs with higher expression changes upon SCI and with CPE. (A,B) Density curves showing distribution of expression changes in total and polysome-bound RNA upon SCI of transcripts containing AREs in the 3′ UTR. (C) Number of transcripts harboring CPE and AREs in the whole transcriptome. (D) Density curves showing the distribution of expression changes of transcripts containing both AREs and CPE in the 3′UTR and those that do not in total and polysome-bound RNA upon SCI. Ticks on top and below the plots represent values of log2 (fold change) of individual transcripts. Distributions were compared with Kolmogorov-Smirnov test. [file Image5.PDF]

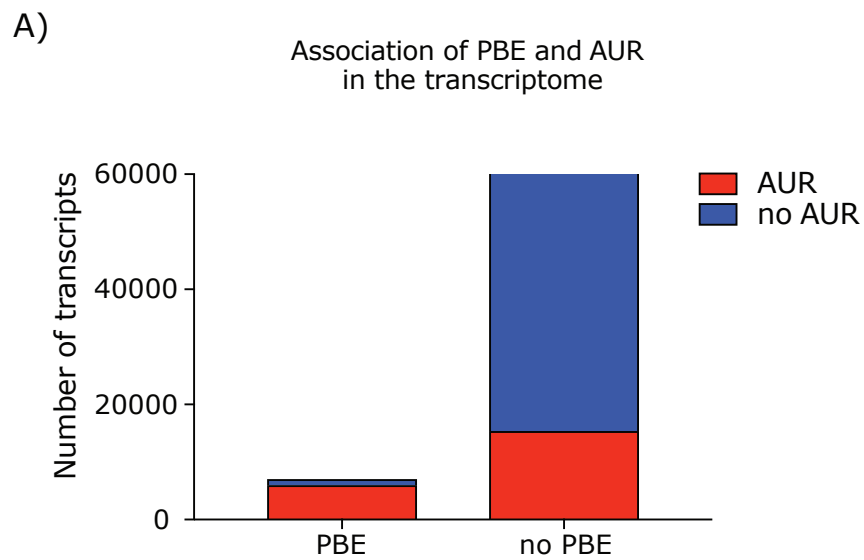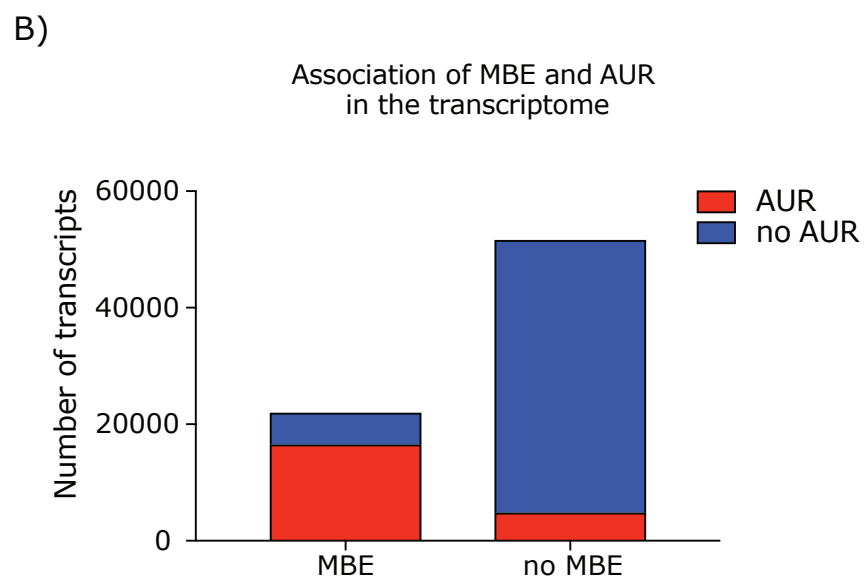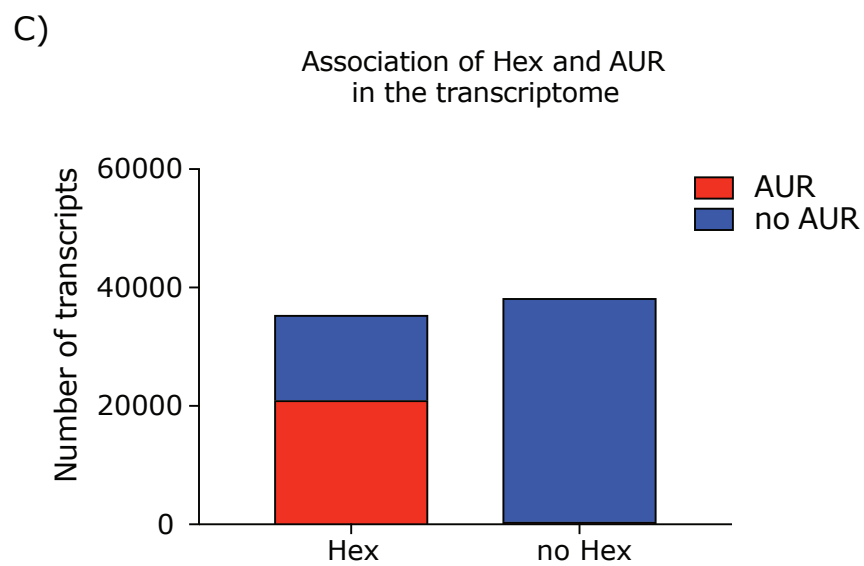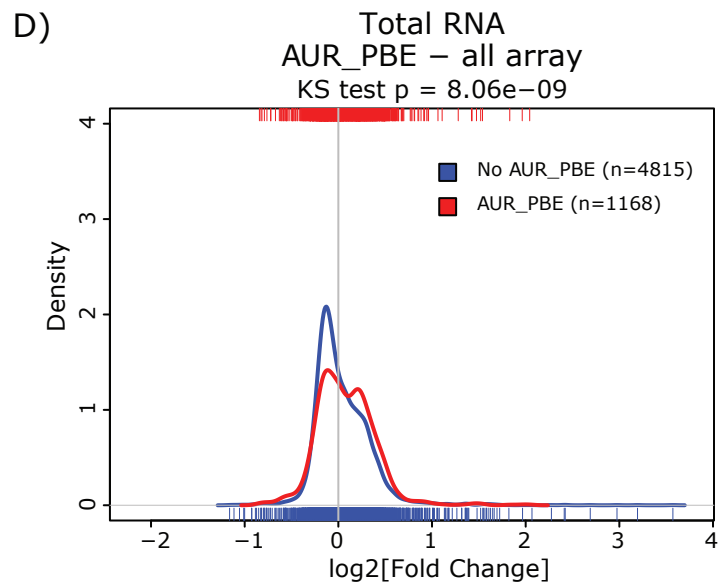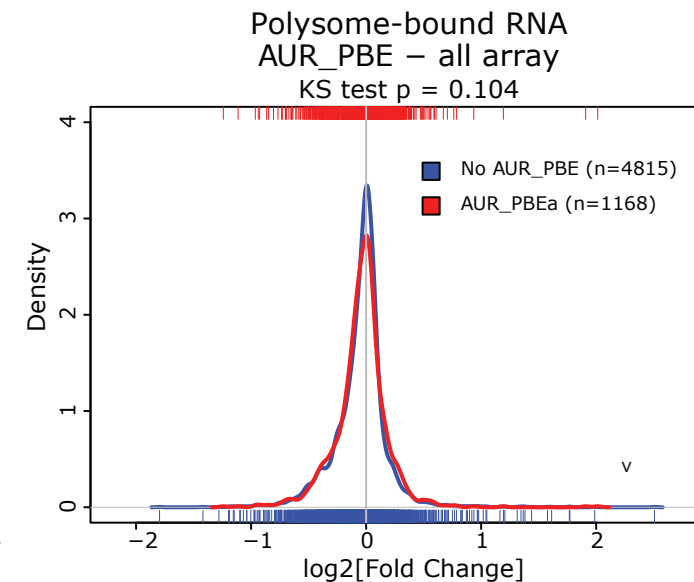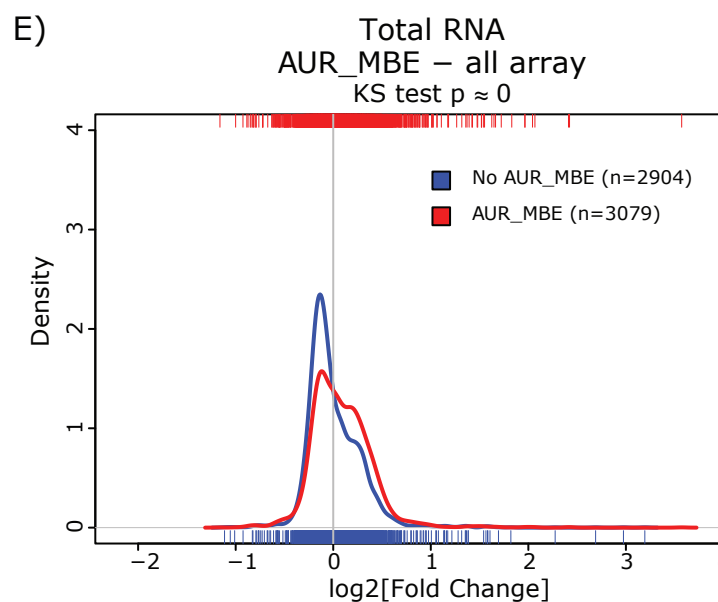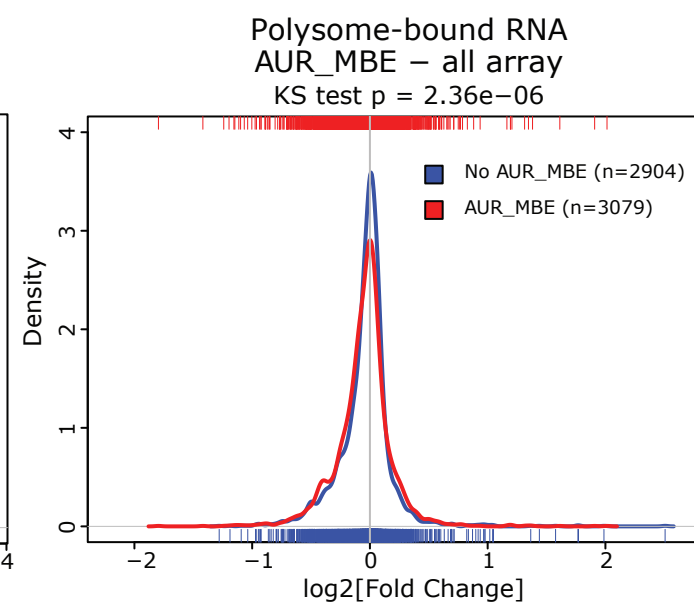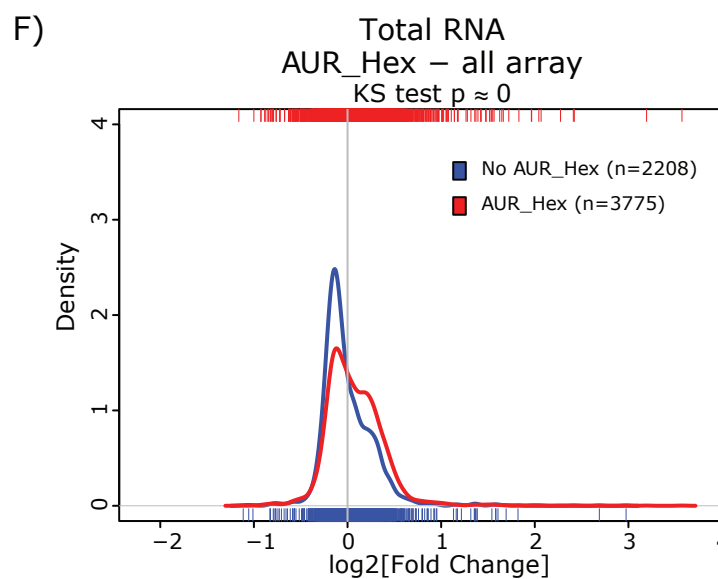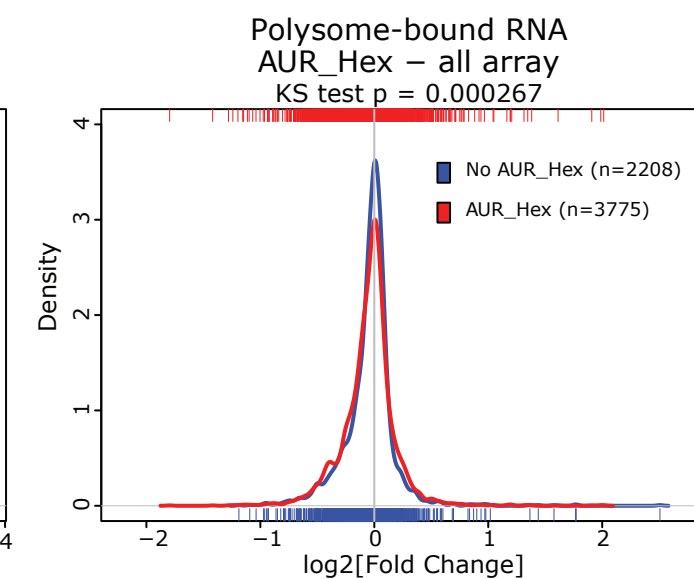

Supplement: Figure S6 — Association of AREs with PBE, MBE, and Hex and higher expression changes upon SCI. (A–C) Number of transcripts harboring AREs and PBE, MBE, or Hex in the whole transcriptome. (D–F) Density curves showing the distribution of expression changes of transcripts containing both AREs and PBE, MBE, or Hex in the 3'UTR and those that do not in total and polysome-bound RNA upon SCI. Ticks on top and below the plots represent values of log2 (fold change) of individual transcripts. Distributions were compared with Kolmogorov-Smirnov test. [file Image6.PDF]

A)

## Total RNA fraction

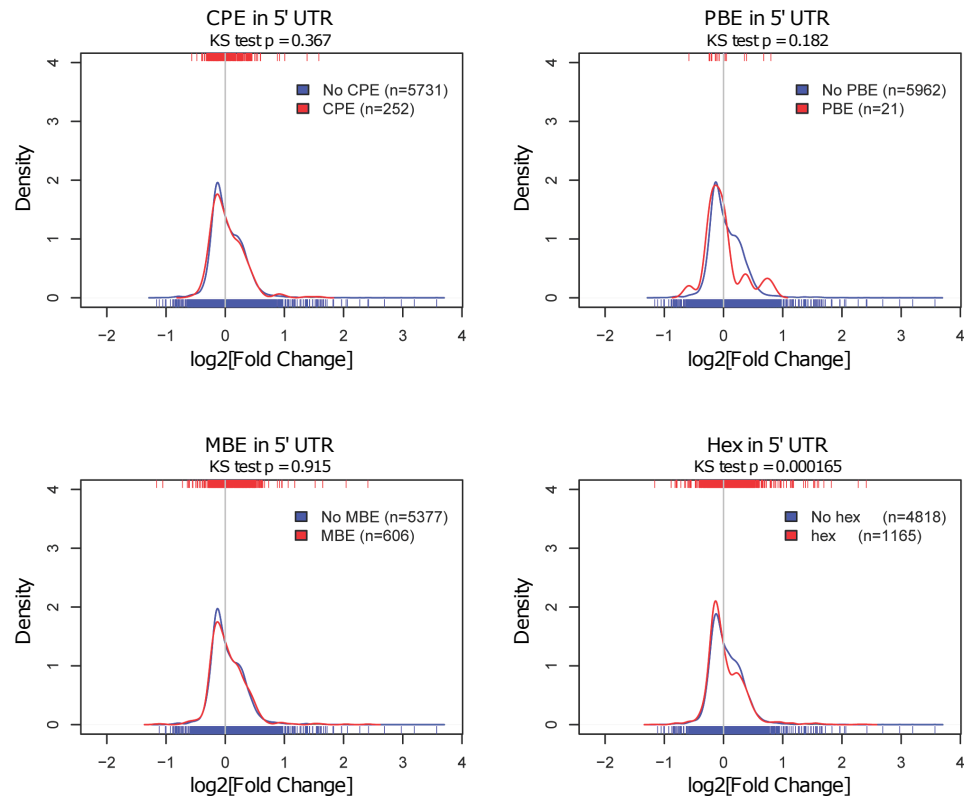

B)

## Total RNA fraction

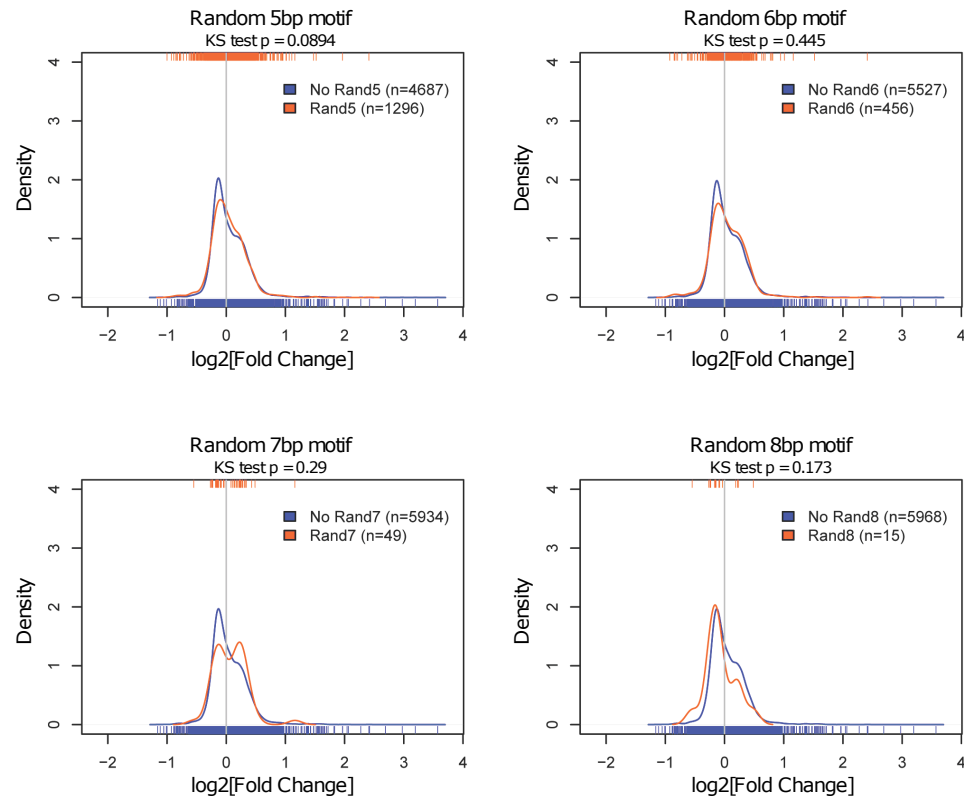

Supplement: Figure S7 — Control analysis for motif analysis. Substituting the motif analysis with (A) the same motifs but in the 5′ UTR and (B) random motifs shows no association with expression changes. [file Image7.PDF]

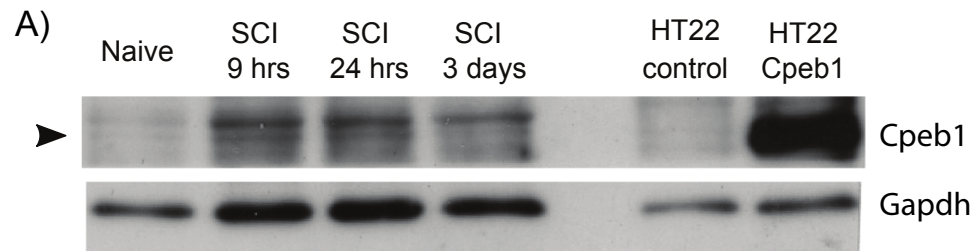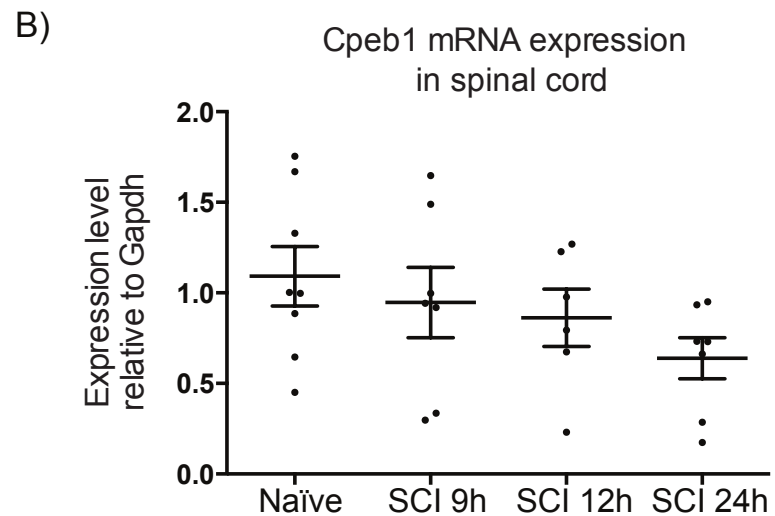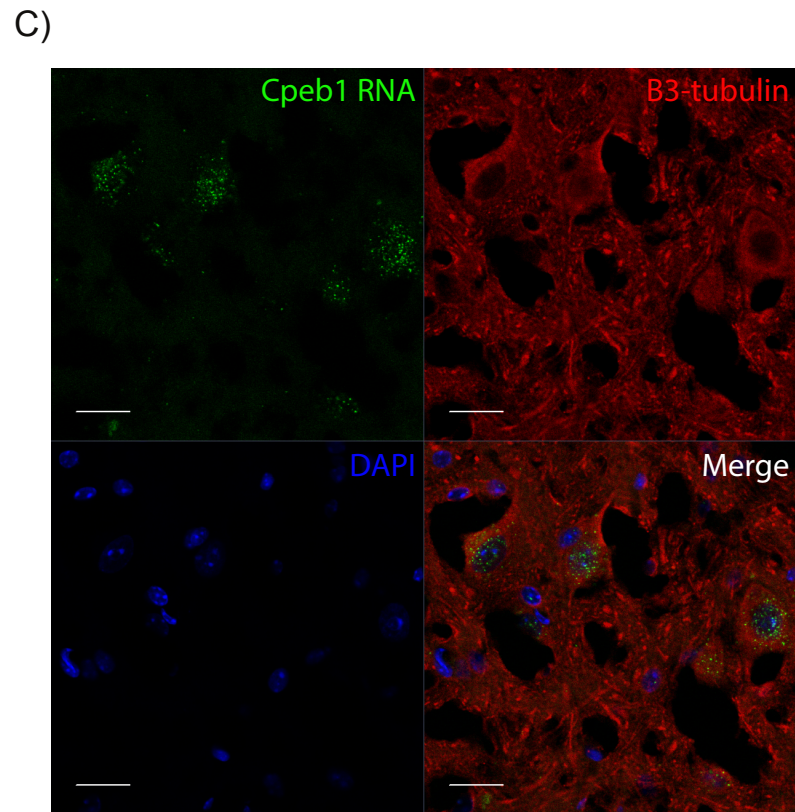

Supplement: Figure S8 — Expression of Cpeb1 in spinal cord tissue. (A) Western blotting of Cpeb1 of naïve and injured spinal cords. Cell lysates of HT22 cells transiently over-expressing Cpeb1 were used as positive control. (B) qPCR of naïve and injured spinal cords for Cpeb1. (1-way ANOVA p = 0.9622). Error bars: mean± S.E.M. (C) Combined FISH and immunohistochemical staining for Cpeb1 RNA and B3-tubulin protein in naïve mouse spinal cord. Note that Cpeb1 transcript expression is restricted to neurons. Scale bars: 20 μm. [file Image8.PDF]

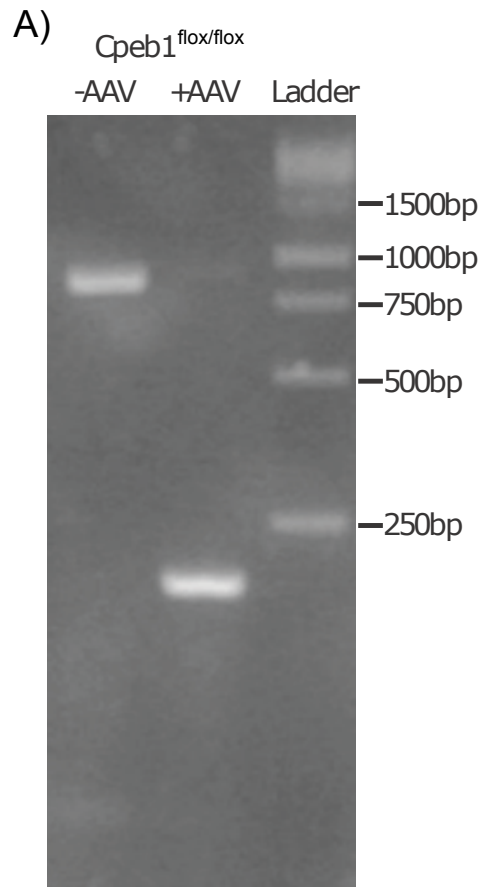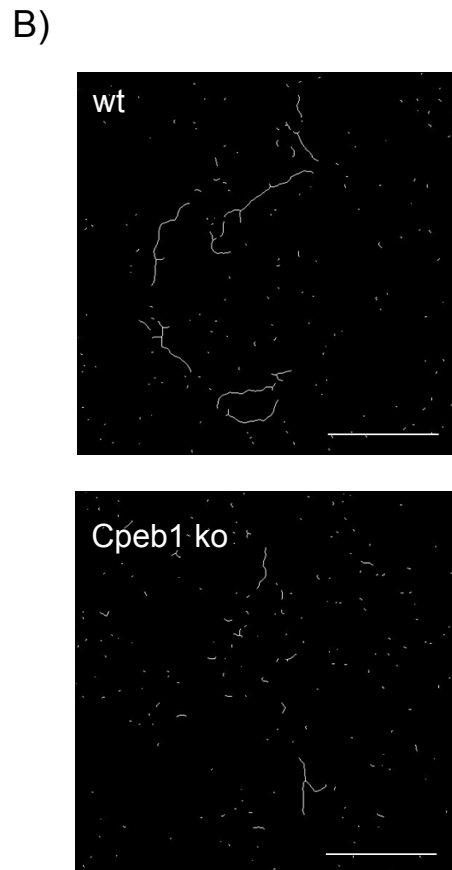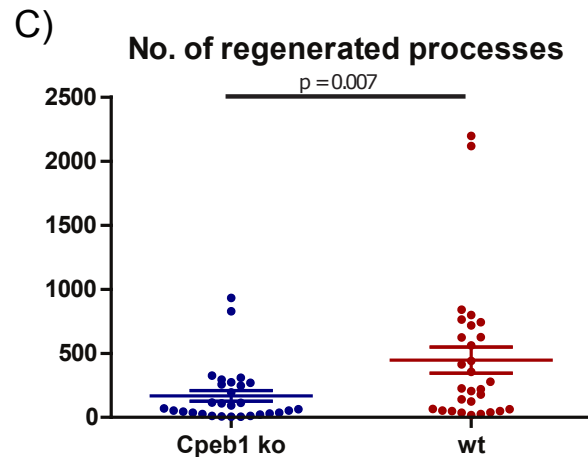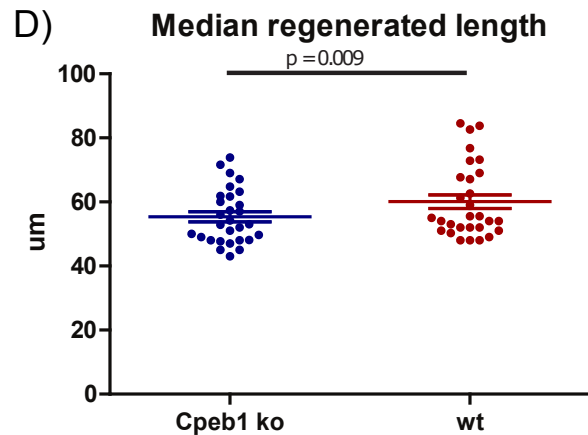

Supplement: Figure S9 — Deletion of Cpeb1 reduces neurite regeneration in vitro. (A) Efficient deletion of Cpeb1 by AAV-delivered Cre confirmed by PCR. Expected sizes: 884 bp (without deletion), 186 bp (with deletion). (B) Cortical neurons infected with AAV-Cre were seeded on transwell chambers, which exclusively allow neurite growth on the lower side of the membrane. Representative images of neurites skeletonized from image processing. (C,D) Quantification of regenerating neurites 24 h after injury. Each data point represents one culture chamber. A total of 29 culture chambers prepared from four mice were used per group. Cpeb1 ko: Cpeb1flox/flox + AAV; wt: wild-type + AAV. Scale bars: 100 μm. Error bars: mean ± S.E.M. [file Image9.PDF]
