# Supplementary material for: Regulation of Adult CNS Axonal Regeneration by the Post-transcriptional Regulator Cpeb1
Source: Front Mol Neurosci. 2018 Jan 12;10:445. doi: 10.3389/fnmol.2017.00445 (PMC5770975; doi:10.3389/fnmol.2017.00445)
Supplement: Figure S4 — Association of PBE, MBE, and Hex with expression changes in transcripts from axon and CNS development GO categories upon SCI. Density curves of fold changes in total (A) and polysome-bound (B) RNA fractions of genes associated with GO categories of axon and CNS development, separated by those that contains PBE, MBE, or Hex and those that do not. There are no transcripts that do not contain Hex under axon development GO category. Ticks on top and below the plots represent values of log2 (fold change) of individual transcripts. Distributions were compared with Kolmogorov-Smirnov test. [file Image4.PDF]

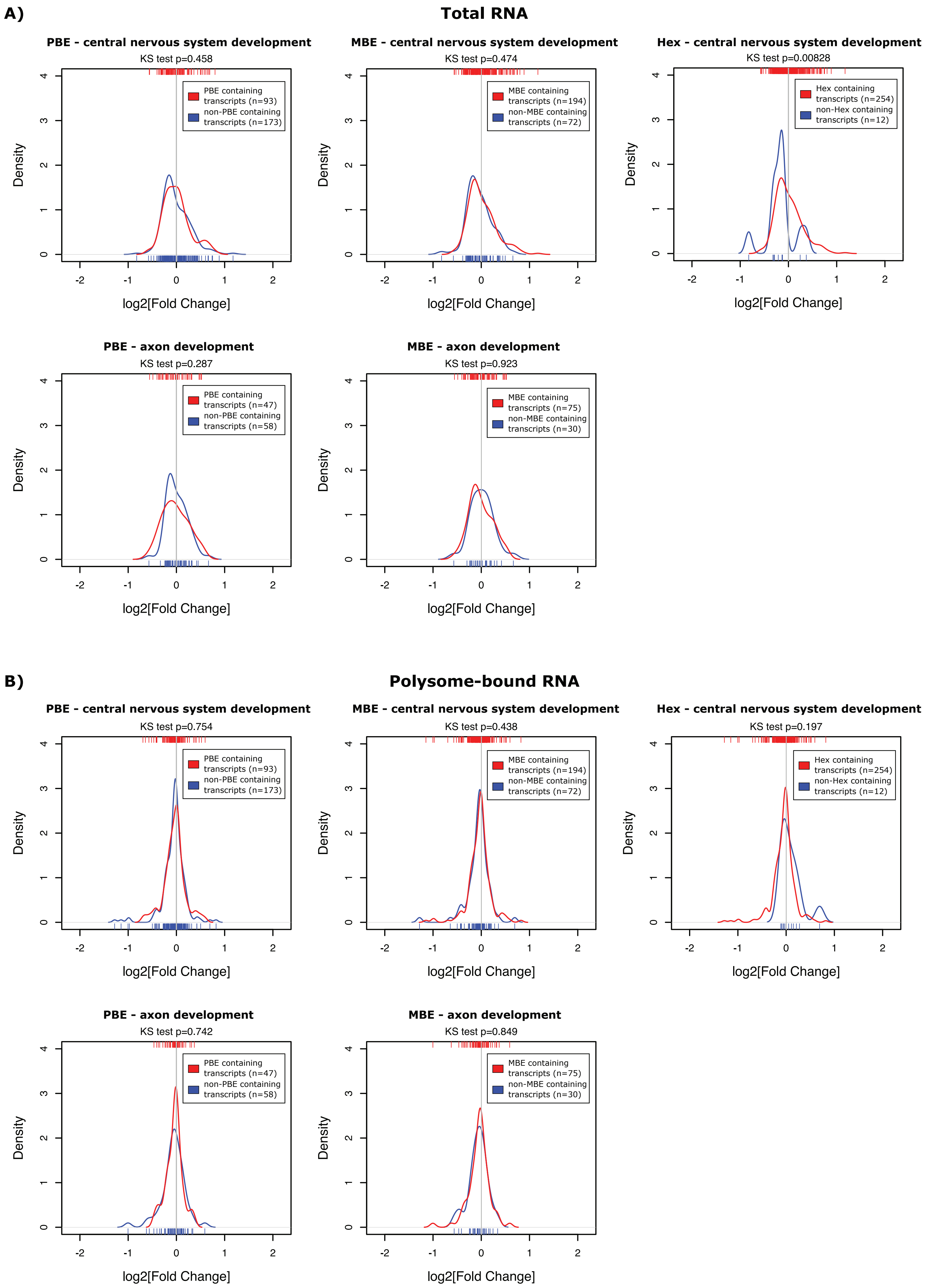
A)
Total RNA
PBE - central nervous system development
KS test p=0.458
PBE containing transcripts (n=93)
non-PBE containing transcripts (n=173)
MBE - central nervous system development
KS test p=0.474
MBE containing transcripts (n=194)
non-MBE containing transcripts (n=72)
Hex - central nervous system development
KS test p=0.00828
Hex containing transcripts (n=254)
non-Hex containing transcripts (n=12)
PBE - axon development
KS test p=0.287
PBE containing transcripts (n=47)
non-PBE containing transcripts (n=58)
MBE - axon development
KS test p=0.923
MBE containing transcripts (n=75)
non-MBE containing transcripts (n=30)
B)
Polysome-bound RNA
PBE - central nervous system development
KS test p=0.754
PBE containing transcripts (n=93)
non-PBE containing transcripts (n=173)
MBE - central nervous system development
KS test p=0.438
MBE containing transcripts (n=194)
non-MBE containing transcripts (n=72)
Hex - central nervous system development
KS test p=0.197
Hex containing transcripts (n=254)
non-Hex containing transcripts (n=12)
PBE - axon development
KS test p=0.742
PBE containing transcripts (n=47)
non-PBE containing transcripts (n=58)
MBE - axon development
KS test p=0.849
MBE containing transcripts (n=75)
non-MBE containing transcripts (n=30)
Density
log2[Fold Change]
