# Supplementary material for: Regulation of Adult CNS Axonal Regeneration by the Post-transcriptional Regulator Cpeb1
Source: Front Mol Neurosci. 2018 Jan 12;10:445. doi: 10.3389/fnmol.2017.00445 (PMC5770975; doi:10.3389/fnmol.2017.00445)
Supplement: Table S5 — List of sequences used in motif analysis. [file Table5.docx]

| CPE | any of the following |
| --- | --- |
|  | TTTTAT |
|  | TTTTAAT |
|  | TTTTACT |
|  | TTTTAA[AG]T |
|  | TTTTCAT |
| PBE | TGTA[ACGT]ATA |
| MBE | [GA]T{1,3}AGT |
| Hex | any of the following |
|  | AATAAA |
|  | ATTAAA |
|  | TATAAA |
|  | AGTAAA |
|  | AAGAAA |
|  | AATATA |
|  | AATACA |
|  | CATAAA |
|  | GATAAA |
|  | AATGAA |
|  | TTTAAA |
|  | ACTAAA |
|  | AATAGA |
| AUR1 | ATTTA |
| AUR2 | TATTTAT |
| AUR3 | TTATTTATT |
| AUR4 | TTATTTA[TA][TA] |
| AUR_CPE | any AUR + any CPE |
| AUR_PBE | any AUR + PBE |
| AUR_MBE | any AUR + MBE |
| AUR_Hex | any AUR + any Hex |
| Random motif 5bp | AGGCG |
| Random motif 6bp | AGGCGT |
| Random motif 7bp | AGGCGTA |
| Random motif 8bp | AGGCGTAA |
